# Fluid shear stress induces a shift from glycolytic to amino acid pathway in human trophoblasts

**DOI:** 10.1186/s13578-023-01114-3

**Published:** 2023-09-08

**Authors:** Beatrice Anna Brugger, Lena Neuper, Jacqueline Guettler, Désirée Forstner, Stefan Wernitznig, Daniel Kummer, Freya Lyssy, Julia Feichtinger, Julian Krappinger, Amin El-Heliebi, Lilli Bonstingl, Gerit Moser, Giovanny Rodriguez-Blanco, Olaf A. Bachkönig, Benjamin Gottschalk, Michael Gruber, Olivia Nonn, Florian Herse, Stefan Verlohren, Hans-Georg Frank, Nirav Barapatre, Cornelia Kampfer, Herbert Fluhr, Gernot Desoye, Martin Gauster

**Affiliations:** 1 Division of Cell Biology, Histology and Embryology, Gottfried Schatz Research Center, Medical University of Graz, Neue Stiftingtalstraße 6, OST V, 8010 Graz, Austria; 2 Department of Obstetrics and Gynaecology, Medical University of Graz, Graz, Austria; 3 Center for Biomarker Research in Medicine (CBmed), Graz, Austria; 4 Clinical Institute for Medical and Chemical Laboratory Diagnosis, Medical University of Graz, Graz, Austria; 5 Division of Molecular Biology and Biochemistry, Gottfried Schatz Research Center, Medical University of Graz, Graz, Austria; 6 Experimental and Clinical Research Center, a cooperation between the Max‐Delbrück‐Center for Molecular Medicine in the Helmholtz Association and the Charité-Universitätsmedizin Berlin, Berlin, Germany; 7 Max‐Delbrück‐Center for Molecular Medicine in the Helmholtz Association (MDC), Berlin, Germany; 8 Charité-Universitätsmedizin Berlin, corporate member of Freie Universität Berlin and Humboldt‐Universität zu Berlin, Berlin, Germany; 9 Department of Obstetrics and Gynaecology, Charité-Universitätsmedizin Berlin, corporate member of Freie Universität Berlin and Humboldt‐Universität zu Berlin, Berlin, Germany; 10 Clinic for Obstetrics, Charité Berlin, Berlin, Germany; 11 Department of Anatomy II, LMU Munich, Munich, Germany

**Keywords:** Placenta development, Fluidic shear stress, Trophoblast metabolism

## Abstract

**Background:**

The human placenta, a tissue with a lifespan limited to the period of pregnancy, is exposed to varying shear rates by maternal blood perfusion depending on the stage of development. In this study, we aimed to investigate the effects of fluidic shear stress on the human trophoblast transcriptome and metabolism.

**Results:**

Based on a trophoblast cell line cultured in a fluidic flow system, changes caused by shear stress were analyzed and compared to static conditions. RNA sequencing and bioinformatics analysis revealed an altered transcriptome and enriched gene ontology terms associated with amino acid and mitochondrial metabolism. A decreased GLUT1 expression and reduced glucose uptake, together with downregulated expression of key glycolytic rate-limiting enzymes, hexokinase 2 and phosphofructokinase 1 was observed. Altered mitochondrial ATP levels and mass spectrometry data, suggested a shift in energy production from glycolysis towards mitochondrial oxidative phosphorylation. This shift in energy production could be supported by increased expression of glutamic-oxaloacetic transaminase variants in response to shear stress as well as under low glucose availability or after silencing of GLUT1. The shift towards amino acid metabolic pathways could be supported by significantly altered amino acid levels, like glutamic acid, cysteine and serine. Downregulation of GLUT1 and glycolytic rate-limiting enzymes, with concomitant upregulation of glutamic-oxaloacetic transaminase 2 was confirmed in first trimester placental explants cultured under fluidic flow. In contrast, high fluid shear stress decreased glutamic-oxaloacetic transaminase 2 expression in term placental explants when compared to low flow rates. Placental tissue from pregnancies with intrauterine growth restriction are exposed to high shear rates and showed also decreased glutamic-oxaloacetic transaminase 2, while GLUT1 was unchanged and glycolytic rate-limiting enzymes showed a trend to be upregulated. The results were generated by using qPCR, immunoblots, quantification of immunofluorescent pictures, padlock probe hybridization, mass spectrometry and FRET-based measurement.

**Conclusion:**

Our study suggests that onset of uteroplacental blood flow is accompanied by a shift from a predominant glycolytic- to an alternative amino acid converting metabolism in the villous trophoblast. Rheological changes with excessive fluidic shear stress at the placental surface, may disrupt this alternative amino acid pathway in the syncytiotrophoblast and could contribute to intrauterine growth restriction.

**Supplementary Information:**

The online version contains supplementary material available at 10.1186/s13578-023-01114-3.

## Background

During human pregnancy, the placenta fulfills a wide range of pregnancy-maintaining functions, including not only the secretion of pregnancy specific hormones, such as human chorionic gonadotropin (hCG), but also regulation of water balance and exchange of gases and metabolites between the maternal and fetal blood circulation [1]. A key cell type of the placenta is the trophoblast—the outer cell mass of the blastocyst—which differentiates into different subpopulations and phenotypes [2, 3]. The so-called villous trophoblast population, including villous cytotrophoblasts (CTB) and the overlying multinucleated syncytiotrophoblast (SCT), together form an epithelial-like layer that covers the whole placental villous trees. In contrast, the extravillous trophoblast (EVT) population differentiates towards an invasive phenotype and migrates into maternal tissues, with the aim to remodel uterine vessels and glands to connect the maternal blood stream to the developing placenta [4]. However, before uteroplacental blood flow is completely established, EVTs accumulate in uterine spiral arteries and form cellular plugs that largely obstruct flow of maternal blood cells to the early placenta. Nevertheless, a low-velocity maternal blood plasma flow acts on placental villi very early in pregnancy. Only by the end of the first trimester, these EVT plugs dissolve, enabling full establishment of uteroplacental perfusion with maternal blood. Hence, the SCT layer on the surface of placental villi, is exposed to increasing degrees of fluidic shear stress (FSS) from 6 weeks of gestation onwards [5–7] to a full established flow after 12 weeks post conception [8, 9]. Recent computational fluid dynamic simulations suggested that the viscous flow of maternal blood generates a wall shear stress that acts tangentially to the apical SCT membrane and was estimated to be 0.5 to 2.3 dyne/cm^2^ in the third trimester of pregnancy [8]. This low FSS has been shown to affect not only morphological aspects of villous trophoblast differentiation including syncytialization and microvilli formation, but also to dysregulate important transporters, such as the glucose transporter type 1 (GLUT1) [5, 10–12]. Since GLUT1 is the main placental glucose transporter and glucose the main nutrient source through the entire period of gestation [13–16], its deregulation in response to FSS may have serious consequences on pregnancy outcome. In addition to glucose, amino acids serve as another main source of nutrients to support adequate growth of placenta and fetus. Impaired transplacental transfer of these nutrients, for any reason, may result in fetal growth restriction [17].

The mechanisms accounting for altered transplacental nutrient transfer are not completely understood and may include multiple compensatory pathways. However, it has become increasingly acknowledged that uteroplacental blood flow, and consequently FSS, may play a key role in this process.

In this study, we aimed to determine effects of FSS on the metabolism of the SCT, the key component of the placental barrier, exposed to maternal blood flow. Using the differentiated trophoblast cell line BeWo as well as first trimester placental explants, cultured either under static or different fluidic flow conditions, we were able to identify a significant change in the trophoblast transcriptome that induces a novel compensatory amino acid pathway in response to impaired trophoblastic glycolytic activity under FSS.

## Results

### Validation of a fluidic flow culture system for analysis of the impact of FSS on the developing placenta

**Fig. 1:**
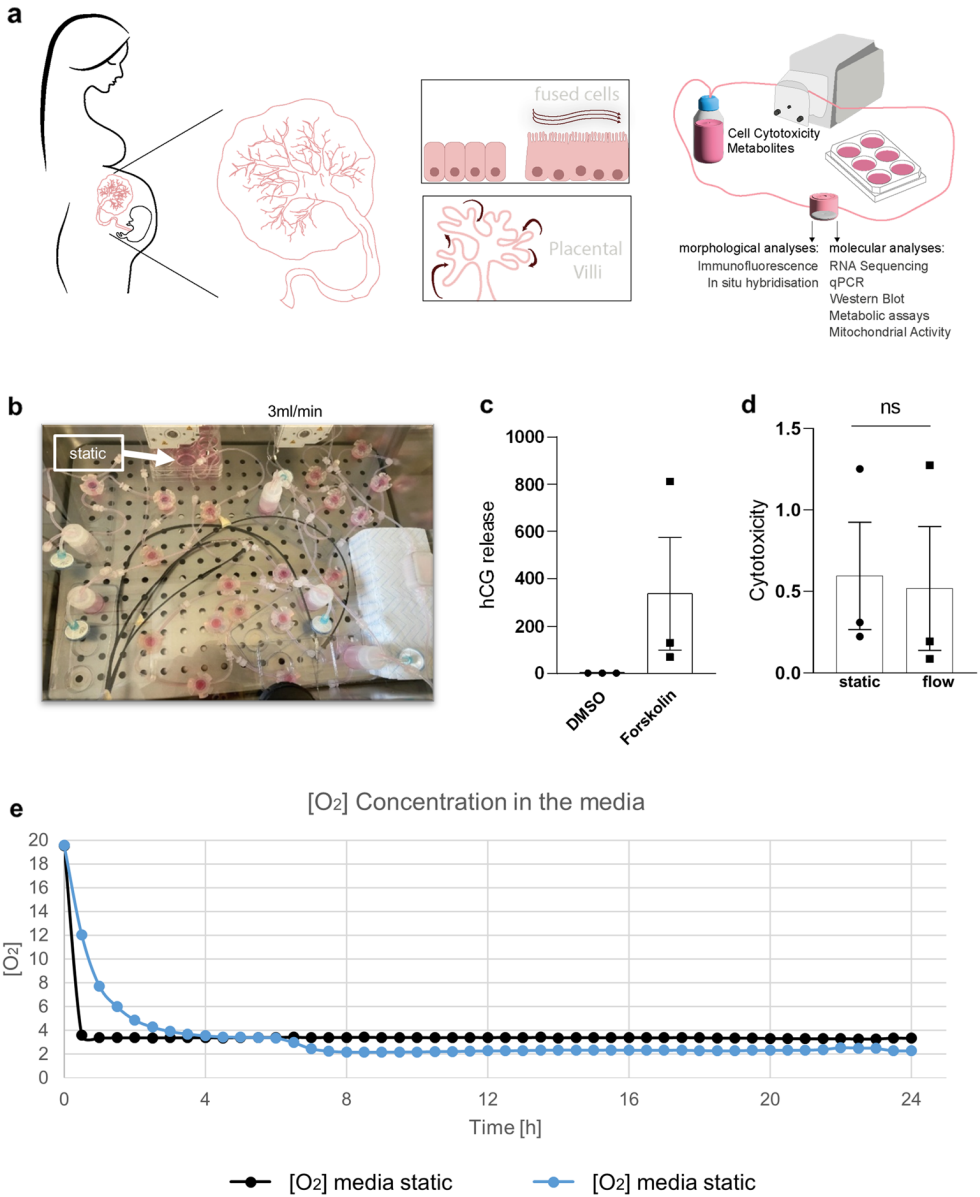
Fluidic flow culture system for analysis of the impact of FSS on the developing placenta. Scheme of the experimental setup illustrates the human placenta, with placental villi bathing in maternal blood flow. The differentiated trophoblast cell line BeWo or human first trimester placental villous explants were cultured under circuit flow in a bioreactor. Downstream analyses after flow culture, including morphological and biomolecular approaches (**a**). Flow chambers were connected to a tubing system and a reservoir bottle. Flow cycles, including 3 chambers each, were run in a TEB500 flow bioreactor. In parallel, cells were cultured in 6-well dishes under static conditions (**b**). Before subjecting cells to fluidic flow culture, forskolin-induced differentiation was confirmed by increased secretion of hCG (**c**). After flow culture, cell viability was determined using a cytotoxicity assay (**d**). Oxygen measurement in the cell culture media of static (black) and flow treated cells (blue) (**e**). Data are presented as mean ± SEM and were obtained from three independent experiments using three different cell passages

In order to study FSS on the SCT in early placenta development, we established a cell- and placental villous explant culture under flow circuit in combination with a physiological low oxygen concentration (Fig. 1a, b and e). For this purpose, the trophoblast cell line BeWo, mimicking a CTB cell line, was first induced with forskolin to undergo differentiation into multinucleated syncytia to form a syncytiotrophoblast like layer [18]. Successful differentiation was confirmed by a strong increase of secreted.

β-hCG (Fig. 1c). Subsequently, cells were either left under static culture or exposed to FSS, which did not negatively affect cell viability as determined by analysis of LDH release and staining for apoptosis marker cleaved caspase 3 (Fig. 1d and Additional file 1: Figure 1). The flow rate was chosen with consideration of physiological shear rates acting on the developing placenta, with a fluidic flow of 3 ml/min (0.95 dyne/cm^2^, see Additional file 1: methods for calculation).

### FSS affects the trophoblast transcriptome

**Fig. 2:**
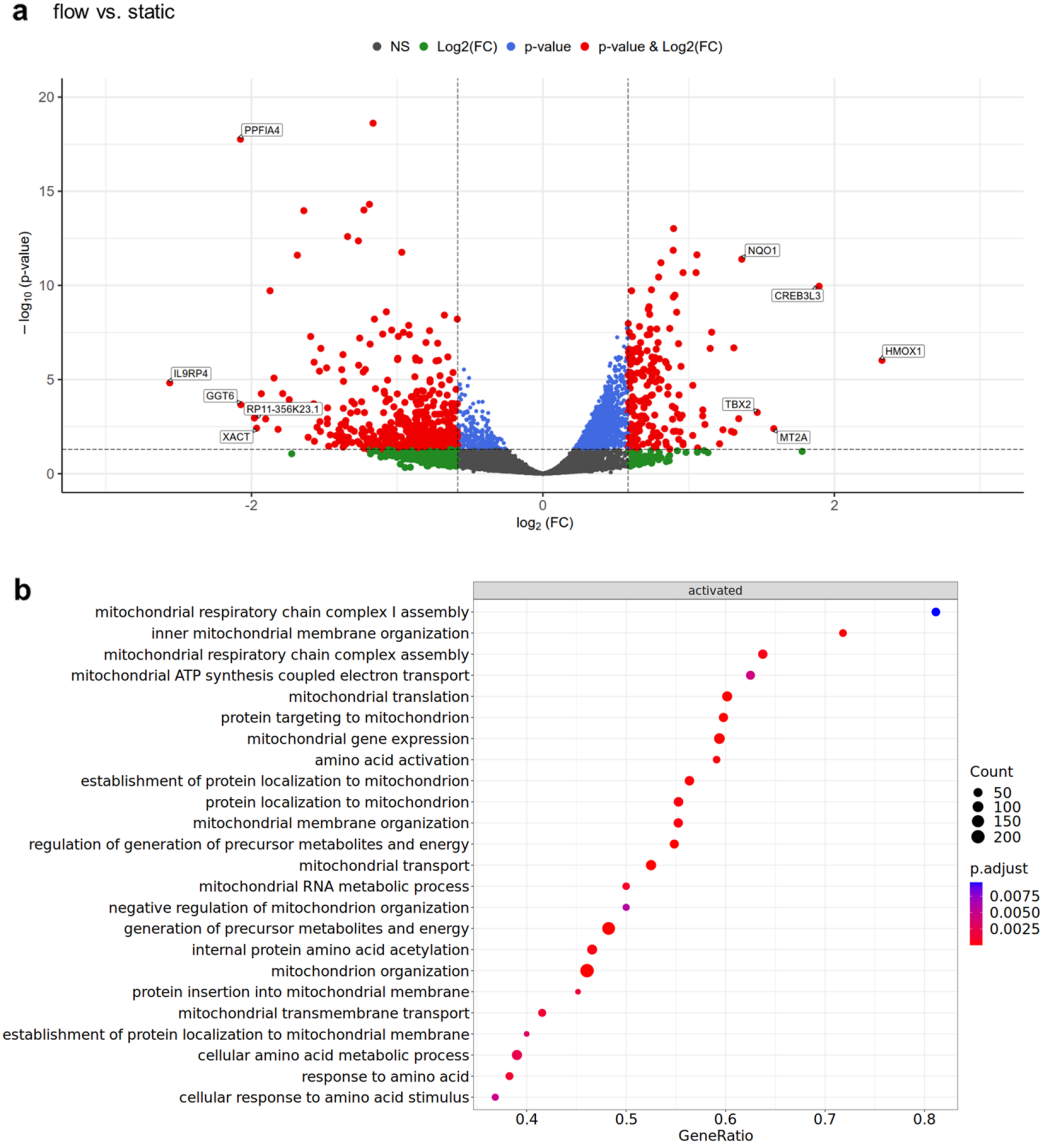
FSS affects the trophoblast transcriptome. The volcano plot showing the significant changes in the transcriptome in differentiated BeWo cells, when cultured under flow, compared to cells cultured under static conditions. Red dots represent significantly differentially expressed genes with an adjusted p-value of ≤ 0.05 and an absolute log2 fold change of ≥ log2(1.5) (**a**). Dot plot showing the GSEA results for relevant gene ontology terms based on the comparison between flow and static conditions. The gene ratio represents the ratio of the number of core enriched genes among all genes. Dots are color-coded depending on their adjusted p-value. Size of the dots are presenting the number of core enriched genes in the respective gene sets (**b**). Presented data are based on triplicates, using three different cell passages each run and are provided in the source data set

RNA-Sequencing (RNA-Seq) and subsequent bioinformatics analysis revealed a significantly altered transcriptome of differentiated BeWo cells that had been cultured under fluidic flow conditions. When compared to static conditions, flow treated cells show a difference in expression of 437 down- and 183 upregulated genes (Fig. 2a, and Additional file 1: Table S1). Remarkably, genes affected by FSS in endothelial cells (EC) show also response in BeWo trophoblast cell line (epithelial phenotype), including upregulation of *HMOX1* [19, 20] and *NQO1* [20] and downregulation of *SLC12A3* [21]. Of note, fluidic flow did not significantly change expression of SCT markers *CSH1*, *ERVW1*, *GATA2, GATA3*, *GDF15*, and *TFAP2A* [3] (Additional file 1: Figure S2 and Suppl. Tab. 1), suggesting only negligible further differentiation of cells under fluidic flow conditions. However, expression of hCG subunits α and β, encoded by *CGA* and *CGB* variants showed a significant increase for *CGA* and many *CGB* family members (*CGB3, CGB5, CGB8*), suggesting a slight flow-mediated modulation of the trophoblastic endocrine activity (Additional file 1: Figure S2 and Additional file 1: Table S1). This is in a good agreement with the increased hCG expression in the first part of pregnancy, until full maternal blood flow is established [22]. Gene set enrichment analysis (GSEA) identified several significant gene ontology (GO) terms, among others associated with amino acid metabolism and mitochondrial metabolism, in cells exposed to fluid shear stress compared to static control (Fig. 2b and Additional file 1: Table S1).

### FSS impairs trophoblastic glucose uptake and expression of genes involved in glycolytic pathway

**Fig. 3:**
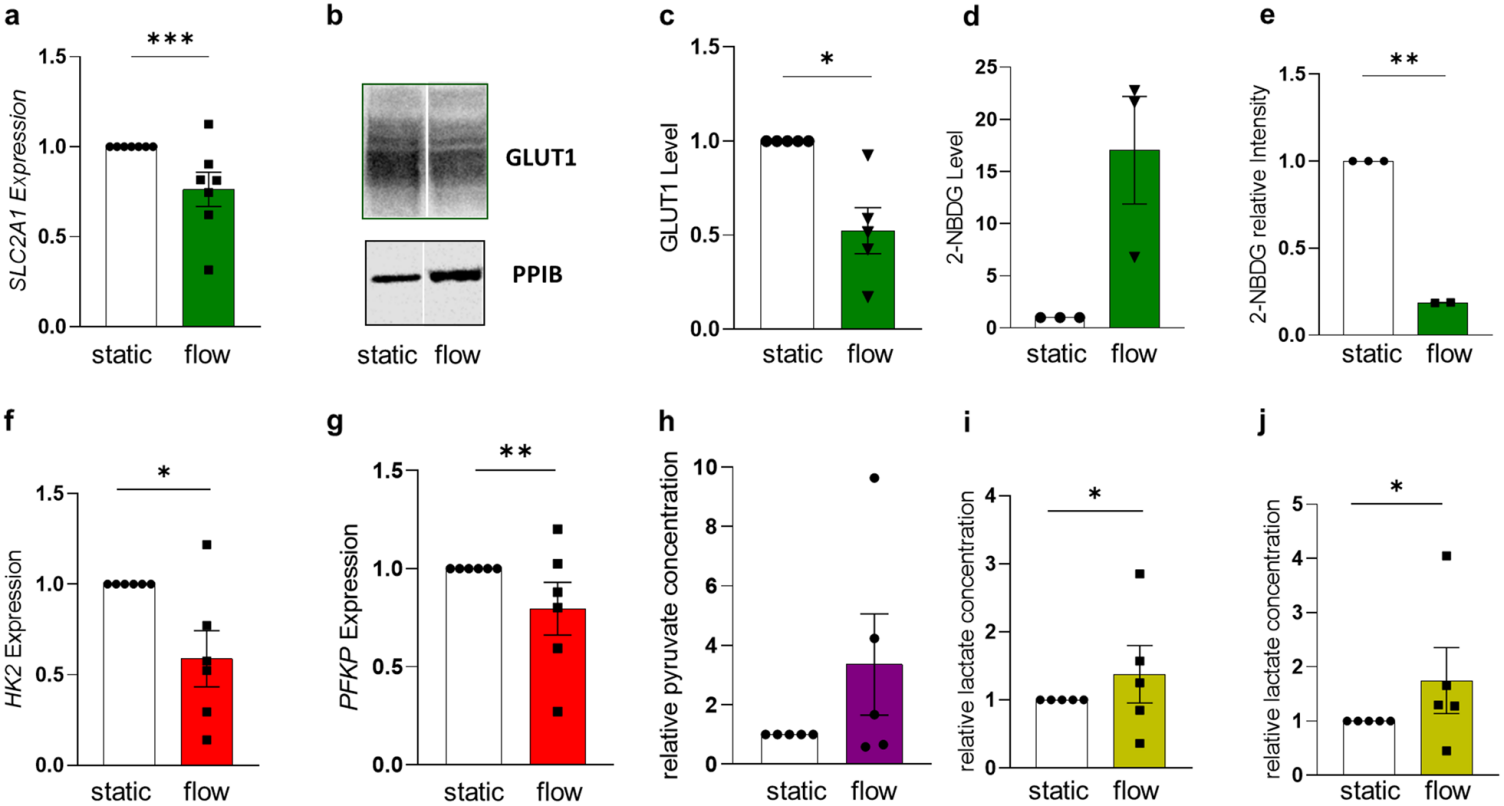
FSS impairs trophoblastic glucose uptake and expression of genes involved in the glycolytic pathway. Cells were cultured either under static or flow conditions for 24 h as indicated. qPCR (**a**) analysis of *SLC2A1* gene expression in cells, a representative Western Blots (**b**) and band densitometry (**c**) for GLUT1 protein. Glucose consumption was determined by 2-NBDG concentration in the supernatant (**d**), and in cell lysates (**e**). qPCR analysis revealed downregulation of *HK2* (**f**) and *PFKP* (**g**) in BeWo cells cultured under flow. Intracellular pyruvate (**h**) as well as intracellular (**i**) and released lactate (**j**) increased in cells in response to fluidic flow. Outlier were detected with Grubbs’ α = 0.05, normal distribution of data was analyzed with Shapiro–Wilk test. Statistical analysis was performed using one sample t-test. Statistical significance was set at p < 0.05. Values represent mean ± SEM based on experiments using three to five different cell passages

Since RNA-Seq data revealed a number of significant gene sets relating to amino acid metabolic pathways, suggesting a general shift in the trophoblast energy homeostasis in response to fluidic flow, we next focused on key factors involved in glucose metabolism (Additional file 1: Figure S3). Analysis of the main placental glucose transporter GLUT1 (encoded by solute carrier family 2 member 1, *SLC2A1*), predominantly located at the apical microvillous plasma membrane of the SCT [17, 23], showed a significant decline of both mRNA (Fig. 3a, p = 0.0002) and protein levels (Fig. 3b and c, p = 0.0128) by up to 50% under fluidic flow conditions, when compared to static controls. To investigate glucose uptake under flow conditions in syncytialized cells, 2-(N-(7-Nitrobenz-2-oxa-1,3-diazol-4-yl)Amino)-2-Deoxyglucose (2-NBDG), supplemented to the culture, remained at higher levels in the supernatant under fluidic flow (Fig. 3d), whereas in undifferentiated BeWo cells the glucose concentration in the supernatant decreased in presence of shear stress (Additional file 1: Figure S4b), confirming previous results by Miura S. et al. [10]. However, in differentiated BeWo cells, intracellular levels of 2-NBDG were decreased compared to static conditions (p = 0.0051, Fig. 3e, Additional file 1: Figure S4a). In addition to decreased GLUT1 levels and reduced glucose uptake in differentiated BeWo cells, expression levels of key glycolytic rate-limiting enzymes, hexokinase 2 (*HK2*, p = 0.0127) and phosphofructokinase 1 (*PFKP*, p = 0.0019) were significantly downregulated under fluidic flow conditions (Fig. 3f and g), overall suggesting less trophoblastic uptake and consumption of glucose in response to increasing FSS. Despite reduced glucose consumption, intracellular pyruvate, the end product of glycolysis, was not significantly changed, but rather indicate a trend to increase in response to fluidic flow (Fig. 3h). Lactate dehydrogenase subunit B (*LDHB*), which preferentially converts lactate to pyruvate [24], was not upregulated under flow (Additional file 1: Figure S4c), and thus rather precluded a conversion from lactate to pyruvate through this pathway. Of note, intracellular (p = 0.0307) and extracellular lactate (p = 0.0455, Fig. 3i and j) increased under flow. Worth mentioning, expression of *PDHX*, encoding component X of the pyruvate dehydrogenase (PDH) complex that links glycolysis to the tricarboxylic acid (TCA) cycle, was deregulated under flow conditions (Additional file 1: Figure S4d).

### FSS influences pathways of energy metabolism and mitochondrial activity in trophoblasts

**Fig. 4:**
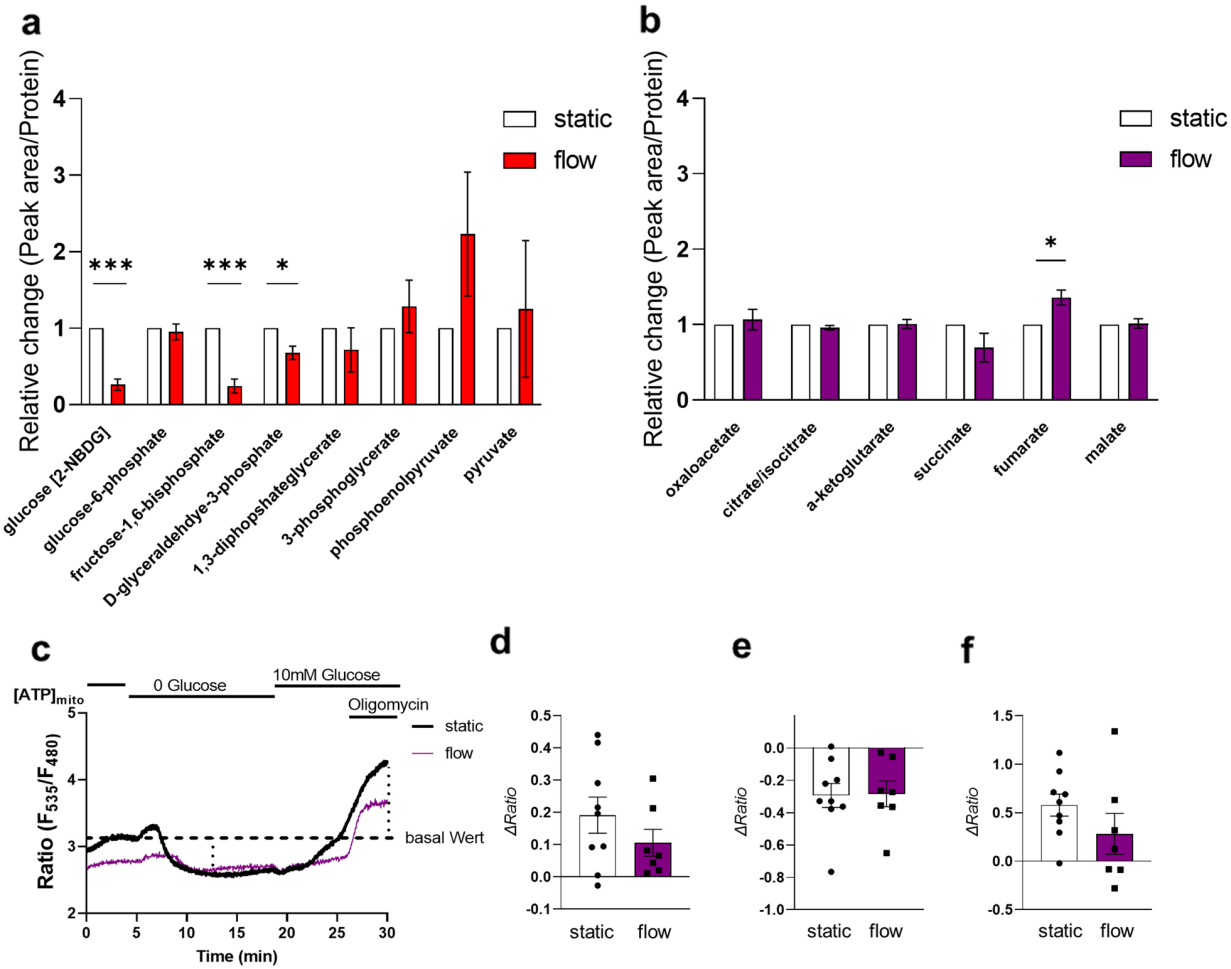
FSS influences pathways of energy metabolism and mitochondrial activity in trophoblasts. Metabolites of the glycolysis (**a**) and the TCA cycle (**b**) are presented for static and flow conditions, representing the peak area normalized to protein content and to the static control. Representative trace of reordered BeWo cells treated either under static (black) or flow (purple) pre-incubation expressing mtAT1.03. Dashed lines indicates the basal ratio used for further calculations. Red dotted lines indicate the deltas quantified (**c**). The maximum value after glucose removal was determined and the basal value subtracted to calculate the delta for hexokinase (**d**). Long-term glucose removal leads to decrease in the ratio (**e**). The minimum was determined before glucose re-addition and basal ratio was substracted. Oligomycin was added to the perfusion after the ratio reached a plateau phase after glucose re-addition. When the ratio reached the plateau under oligomycin the experiment was stopped (**f**),—either minimum or maximum was determined, depending on the cells reaction, and basal substracted (n = cells/days static 9/4; 0.95 dyne/cm^2^ 7/4). Outlier were detected with Grubbs’ α = 0.05, normal distribution of data was analyzed with Shapiro–Wilk test. Statistical analysis for the relative abundance of metabolites was performed with a multiple unpaired t-test. Statistical analysis for the mitochondrial assay was performed using an unpaired t-test. Statistical significance was set at p < 0.05. Values represent mean ± SEM based on experiments using three to five different cell passages

Based on the observed impaired glucose consumption in response to FSS, we next analyzed metabolites of the glycolytic pathway. We were able to detect a significant decrease (p = 0.0002) of fructose-1,6-bisphosphate, the product of fructose-6-phosphate phosphorylation by phosphofructokinase. Additionally, the downstream metabolite glyceraldehyde-3-phosphate showed a significant decrease (p = 0.0103). However, pyruvate levels did not significantly increase, at least by using this method, although a statistical trend for increase could be confirmed (Fig. 4a). Based on the impaired glucose consumption followed by a decrease of the glycolytic pathway and on the results of RNA-Seq, showing a change in the mitochondrial metabolism, we further aimed to assess the metabolites of the TCA cycle. We detected a significant increase of fumarate (p = 0.0114, Fig. 4b). Furthermore, we analyzed the mitochondrial function of cells exposed to FSS. Differentiated BeWo cells showed a highly heterogeneous metabolic setup irrespective of static-, or flow conditions. We measured [ATP]_mito_ in mtAT1.03 expressing BeWo cells. In our protocol, we first removed glucose, followed by glucose re-addition and challenging with oligomycin. The initial elevation of [ATP]_mito_ after glucose removal was shown to be linked to the activity of hexokinase [25] and intracellular glycolytic activity, likewise to the minimal [ATP]_mito_ after continued glucose removal. Re-addition of glucose and challenging with oligomycin reports the capacity of BeWo cells to use oxidative phosphorylation to maintain the mitochondrial membrane potential and produce [ATP]_mito_. Cells using the mitochondrial membrane potential for ATP generation show a reduction of [ATP]_mito_ upon FoF1-ATPase inhibition by oligomycin treatment, while cells using ATP to maintain the membrane potential mediated by a reverse FoF1-ATPase show an increase of [ATP]_mito_ [25] (Fig. 4c). Analysis of mitochondrial activity showed a reduction of hexokinase activity in cells exposed to shear stress. Although it did not reach statistical significance (Fig. 4d), the results were in good agreement with significantly downregulated *HK2* expression. Basal ratio was defined as average ratio of one minute before perfusion was changed to no glucose buffer. The ATP drop during glucose deprivation was not dependent on the pre-treatment of the cells (Fig. 4e). Addition of the FoF1-ATPase inhibitor oligomycin led predominantly to an increase of mitochondrial ATP. These results point to a FoF1-ATPase in reverse mode, using ATP to maintain the mitochondrial membrane potential [25]. Like the initial ATP rise after glucose deprivation, a trend for oligomycin-induced changes was visible of mitochondrial ATP levels. Cells exposed to shear stress showed reduced ATP increase or even a mitochondrial ATP decrease after oligomycin treatment pointing to a FoF1-ATPase in forward mode using the membrane potential to generate ATP (Fig. 4f).

### FSS induces GOT dependent amino acid metabolism in trophoblasts

**Fig. 5:**
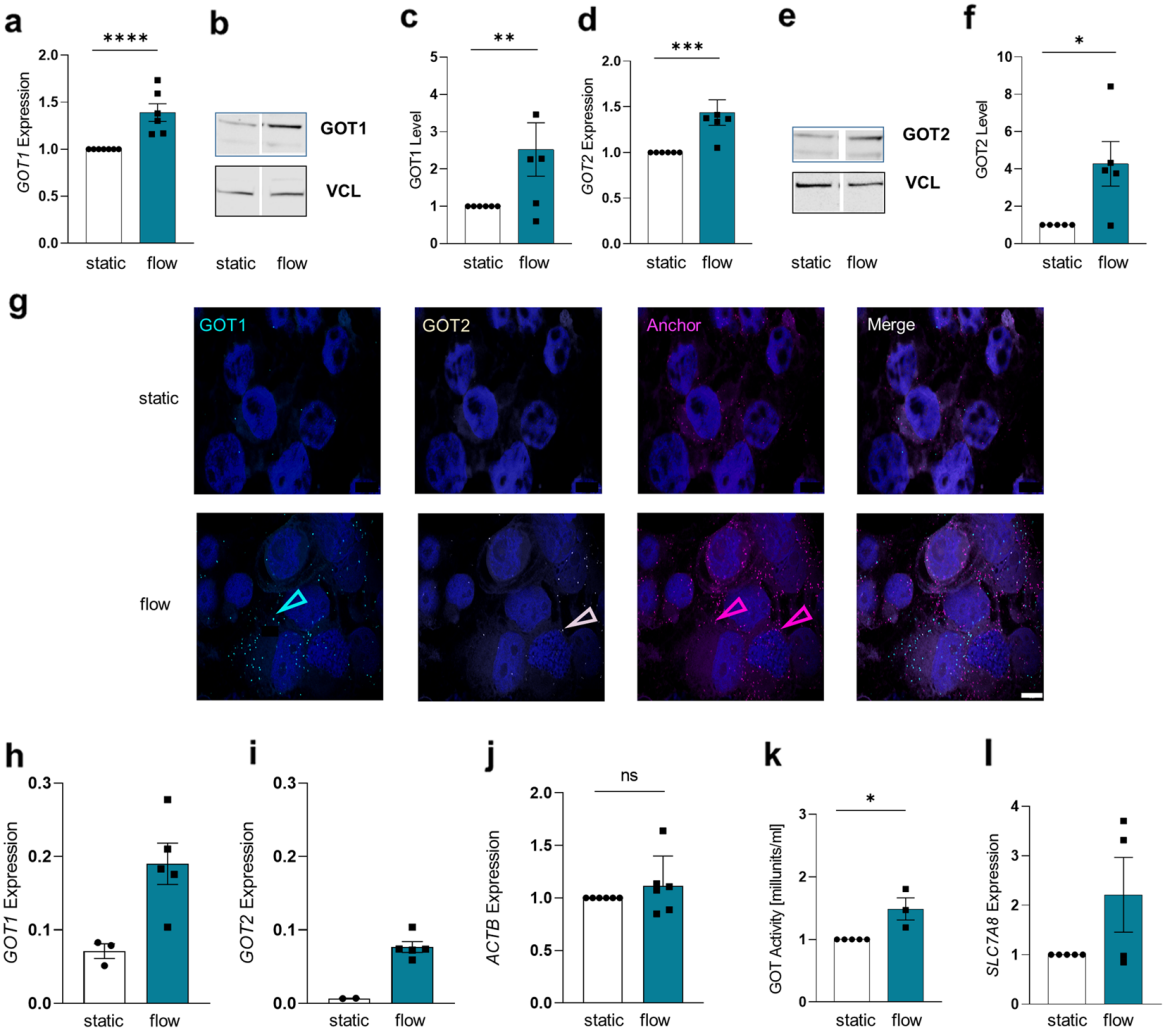
FSS induces GOT-dependent amino acid metabolism in trophoblasts cells were cultured either under static, or flow conditions for 24 h. qPCR analysis of *GOT1* (**a**). Representative Western Blot (**b**) and band densitometry (**c**) showed increasing GOT1 levels in BeWo cells exposed to shear stress. GOT2 mRNA (**d**) and protein levels (**e** and **f**) were upregulated in cells cultured under flow conditions. Padlock probe-based in situ hybridization (**g**) detected GOT1 (blue dots) and GOT2 transcripts (yellow dots) in BeWo cells. The anchor, confirming positive signals, is shown in pink. Scale bar: 20 µM. I*n situ* hybridization analysis represent *GOT1* (**h**) and *GOT2* (**i**). Data were normalized to *ACTB* (**j**). GOT activity (**k**) increased in cells exposed to shear stress compared to static conditions. *SLC7A8* mRNA (**l**) increase in cells exposed to shear stress. Data are presented as mean ± SEM based on experiments using five different cell passages. In situ hybridization was performed three times. Outlier were detected with Grubbs’ α = 0.05, normal distribution of data was analyzed with Shapiro–Wilk test. Statistical analysis was performed using one sample t-test. Statistical significance was set at *p* < 0.05

Since the glycolytic pathway is altered, we aimed to investigate an additional pathway may be supporting glycolysis for energy production in cells cultured under fluidic flow. Since RNA-Seq data suggested a flow dependent upregulation of the cytoplasmic and the inner-membrane mitochondrial isoforms of glutamic-oxaloacetic transaminases, GOT1 and GOT2, respectively, we next validated their expression levels by qPCR and immunoblotting. Both, GOT1 (mRNA expression p < 0.0001; protein level p = 0.0170, Fig. 5a–c) and GOT2 (mRNA expression p = 0.0001; protein level p = 0.0232, Fig. 5d–f) significantly increased in response to shear stress. To identify whether these findings may be translated as a general concept to all cell types exposed to fluidic flow or whether they are specific for epithelial-like cell layers, such as the villous trophoblast, we next analyzed glucose uptake, GLUT1 and *HK2* expression in human umbilical vein endothelial cells (HUVEC). The results suggested, that GLUT1 is not in general downregulated in different cell types exposed to shear stress (Additional file 1: Figure S5a–c) but showed slightly higher glucose concentration in the extracellular compartment as well as a significant downregulation of *HK2* (Additional file 1: Figure S5d, e). GOT1 and GOT2 expression data showed similar results in HUVEC and BeWo cells, by significant upregulation of both GOT enzymes in cells exposed to FSS (Additional file 1: Figure S5f–j). In line with this observation, mRNA-based padlock probe in situ hybridization also confirmed the flow-dependent upregulation of *GOT1* (Fig. 5g and h) and *GOT2* (Fig. 5g and i) with increasing flow in BeWo cells, by using *ACTB* (Fig. 5j) as a reference gene. Notably, increased GOT expression levels were reflected by increased GOT enzyme activity (p = 0.0193, Fig. 5k). At the same time, solute carrier family 7 member 8 (*SLC7A8*), which plays an essential role in the transport of small and large neutral amino acids, showed a trend to be upregulated under fluidic flow (Fig. 5l). Beside glutamic acid and glycine, a broad panel of proteinogenic amino acids, such as asparagine, glutamine and serine, were significantly deregulated in cells cultured under fluidic flow (Additional file 1: Figure S6a). Cysteine, an upstream metabolite for the conversion to pyruvate showed lower concentrations in flow-treated cells on intracellular (p = 0.0113) and extracellular levels (p = 0.0007, Additional file 1: Figure S6b and c). Moreover, genes encoding enzymes for the conversion of substrates for glutathione formation (Additional file 1: Figure S6e) were downregulated in flow treated trophoblasts (*GSS, GCLC*, Additional file 1: Figure S6d and f).

### Dependence of GOT1 and GOT2 from glucose availability

**Fig. 6:**
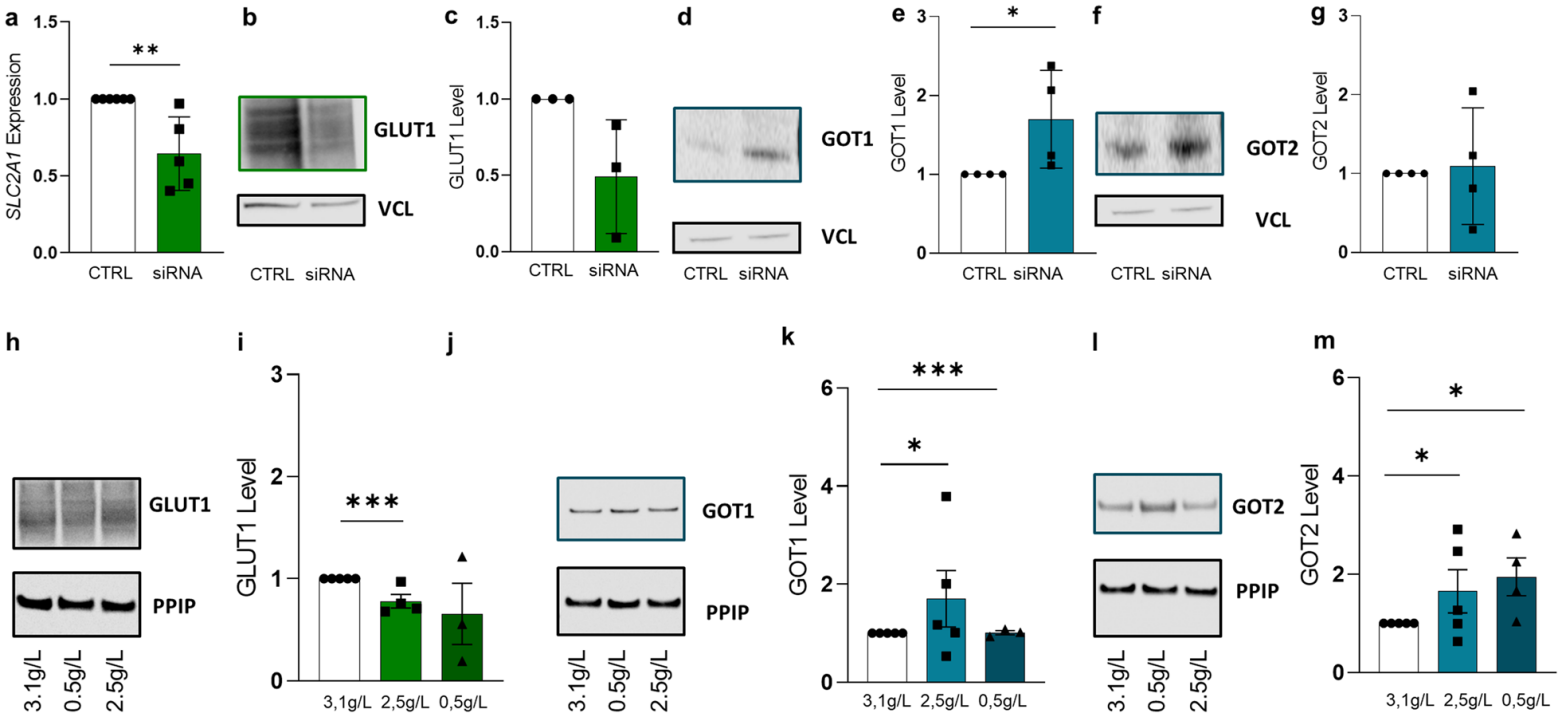
Dependence of GOT1 and GOT2 from glucose availability Efficiency of *SLC2A1* silencing in differentiated BeWo cells was determined for CTRL and silenced samples on mRNA (**a**) and protein level (**b** and **c**). Western Blot (**d**) and band densitometry (**e**) revealed increased GOT1 protein levels, while GOT2 levels (**f** and **g**) remained unchanged in *SLC2A1*-silenced cells. Cells were cultured with three different glucose concentrations. Immunoblotting (**h**) and band densitometry (**i**) showed declining GLUT1 levels with decreasing available glucose concentrations. GOT1 (**j** and **k**) and GOT2 (**l** and **m**) levels increased when cultured in low glucose medium. Experiments were performed a minimum of three times. Outlier were detected with Grubbs’ α = 0.05, normal distribution of data was analyzed with Shapiro–Wilk test. Statistical analysis was performed using one sample t-test for the protein analysis. Statistical significance was set at *p* < 0.05

To identify a potential link between the glycolytic pathway and a possible shift to the amino acid pathway, we next silenced the main glucose transporter GLUT1 (encoded by *SLC2A1*, mRNA expression p = 0.0039, Fig. 6a–c). Of note, silencing of GLUT1 led to a downregulation of *HK2*, suggesting a general restriction of the glycolytic pathway (p = 0.0032, Additional file 1: Figure S7a), while at the same time GOT1 levels significantly increased (Fig. 6d, e, p = 0.0119). In contrast, GOT2 enzyme expression was not affected from GLUT1 silencing (Fig. 6f, g). As glucose may enter the cell with diffusion, we next performed experiments with different glucose availability for the cells. Cultivation in low glucose medium led to downregulation of GLUT1 (p = 0.0013, Fig. 6h, i), as well as decreased *HK2* (p = 0.018 and 0.005) and *PFKP* (p = 0.0065 and 0.001) expression in BeWo cells (Additional file 1: Figure S7b and 7c). With lower glucose availability, GOT1 increased (p = 0.0411), however, with 0.5 g/l glucose in the culture media it was decreased (p = 0.0018). GOT2 levels significantly increased (p = 0.015, Fig. 6j–m), suggesting that GOT2 supports the glycolysis by maintaining the energy balance in BeWo cells through promoting the amino acid pathway in a situation of glucose shortage.

### FSS affects energy metabolism in first trimester explants

**Fig. 7:**
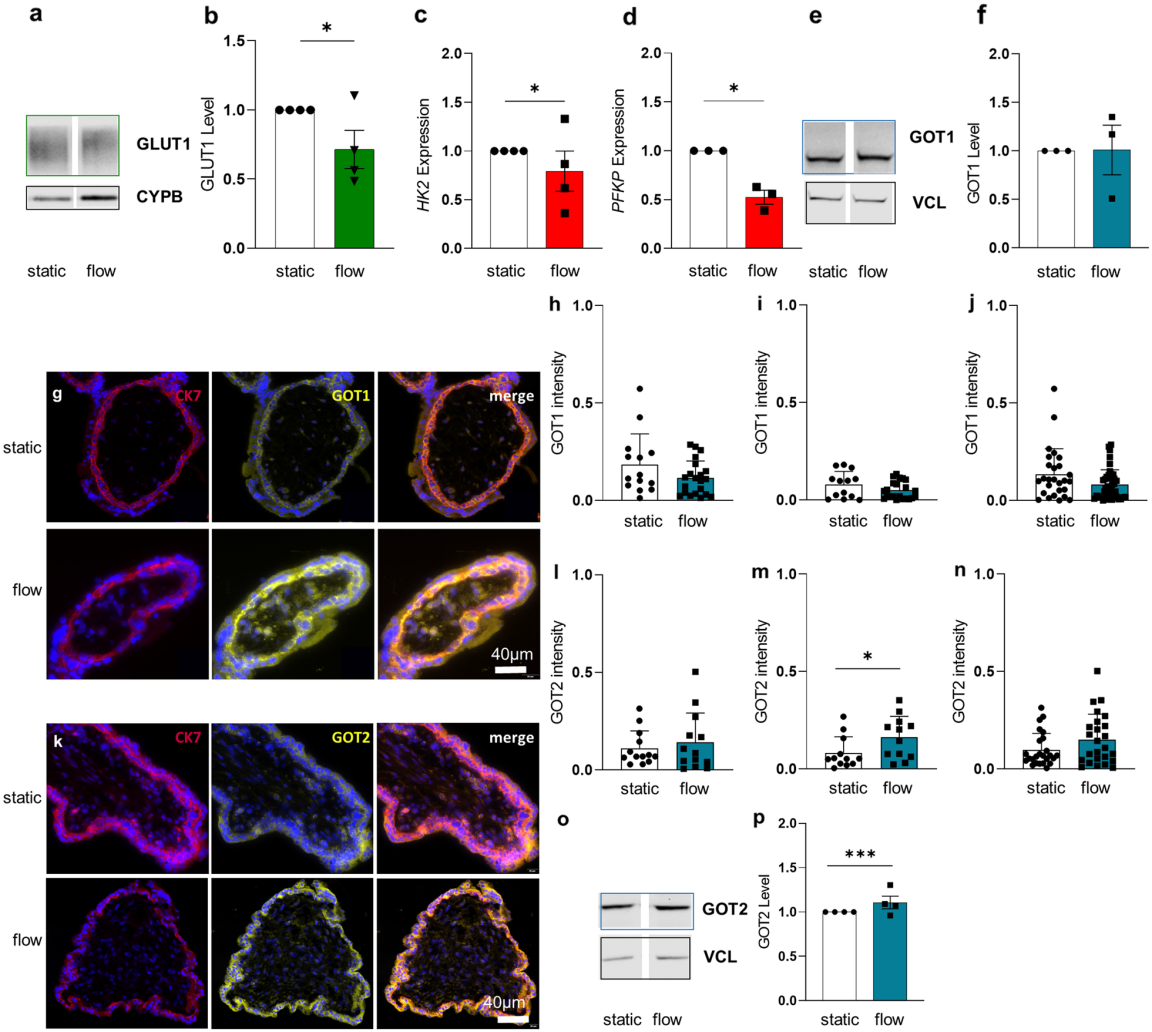
FSS affects GLUT1, GOT1 and GOT2 expression in different trophoblast layers. First trimester villi were cultured either under static or flow conditions for 24 h. Western Blot for GLUT1 receptor in first trimester placental villi (**a**) and related band densitometry (**b**). Key glycolytic enzymes *HK2* (**c**) and *PFKP* (**d**) expression levels Representative Western Blot (**e**) and band densitometry (**f**) showed no change in placental GOT1 levels. Representative immunofluorescence staining of human placental first trimester explant cultures stained for CK7 (red, upper panel), GOT1 (yellow) (**g**). Nuclei were stained with DAPI. Software-based image analysis showed downregulation of GOT1 in the CT (**h**), as well as in the SCT layer (**i**), and total villous trophoblast compartment (**j**) under fluidic flow. Immunofluorescence staining of first trimester placental villi for CK7 (red), and GOT2 (yellow) (**k**). Software-based image analysis showed no significant change of GOT2 intensity in the CT (**l**), whereas GOT2 intensity increased in the SCT (**m**) and the total villous trophoblast (**n**) under fluidic flow. Representative Western Blot (**o**) and band densitometry (**p**) showed GOT2 upregulation under flow conditions in placental explants. Immunofluorescence and Western Blot analysis were performed with four different placental samples. Outlier were detected with Grubbs’ α = 0.05, normal distribution of data was analyzed with Shapiro–Wilk test. Statistical analysis was performed using one sample t-test for the protein analysis. Statistical analysis for the immunofluorescent staining was performed with an unpaired t-test. Statistical significance was set at *p* < 0.05. Values represent mean ± SEM.

Next, we sought to confirm the effect of fluidic flow on the expression of GLUT1, GOT1 and GOT2 in first trimester placental explants, exposed to same conditions as applied for BeWo cells. This revealed decreased GLUT1 levels (p = 0.0139) showed by immunoblot data (Fig. 7a, b), as well as a significantly decreased expression of the key enzymes of the glycolysis *HK2* (p = 0.0309) and *PFKP* (p = 0.0182, Fig. 7c, d). Total placental GOT1 levels did not change in response to FSS (Fig. 7e, f). However, expression of GOT1 seems to be predominantly localized in villous CTs of first trimester placental explants (Fig. 7g), nevertheless, immunofluorescence intensity decreased in trophoblasts in flow culture. (Fig. 7h–j). However, an opposite spatial distribution was observed for GOT2, which was predominantly detected in the SCT (p = 0.0449, Fig. 7k and m) and further increased in explants subjected to fluidic flow (Fig. 7n). GOT2 was not significantly upregulated in CTs (Fig. 7l), whereas total placental GOT2 levels increased under flow conditions (p = 0.0006, Fig. 7o, p).

### GLUT1 and GOT2 expression in pregnancies complicated by intrauterine growth restriction

**Fig. 8:**
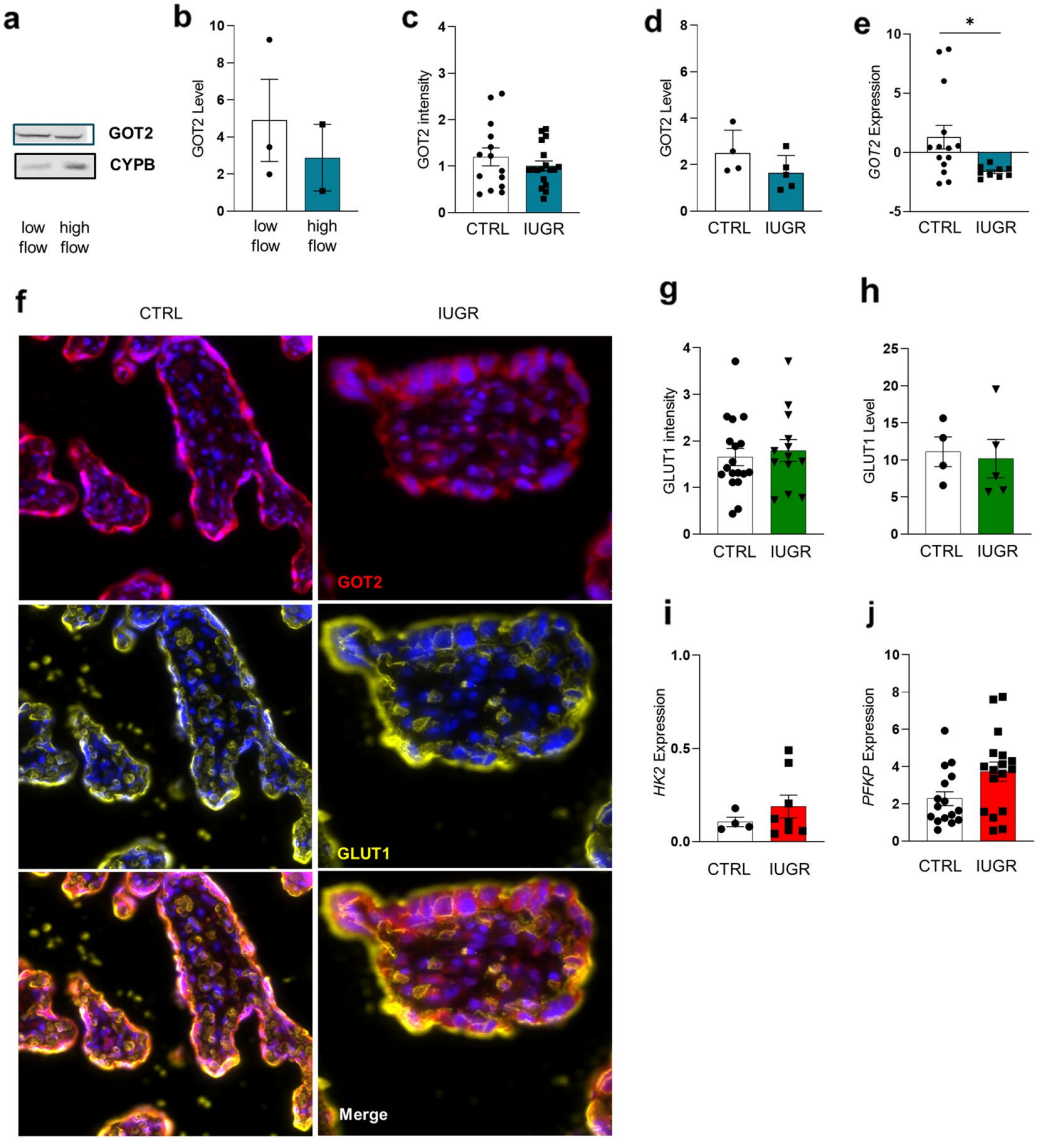
GLUT1 and GOT2 expression in pregnancies complicated by intrauterine growth restriction. Western Blot of GOT2 level in term placental explants exposed either to low or high FSS (**a**) and band densitometry (**b**). GOT2 intensity in IUGR placentas (**c**) and the representative staining for data shown in (**f**). Western blot (**d**) and qPCR (**e**) of placental GOT2 levels in IUGR and control cases. Analyses were performed with a minimum of five placenta samples for Western Blot, nine IUGR placenta samples for qPCR, and 20 different IUGR placenta for IF. Immunofluorescence picture represents GOT2 (red) and GLUT1 (yellow). Nuclei are stained with DAPI (**f**). GLUT1 expression is not significantly affected in IUGR placenta compared to control in all analysis (**g** and** h**). Key enzymes of the glycolytic pathway *HK2* (**i**) and *PFKP* (**j**) are slightly upregulated in IUGR compared to CTRL placentas. Outlier were detected with Grubbs’ α = 0.05, normal distribution of data was analyzed with Shapiro–Wilk test. Statistical analysis was performed using one sample t-test for the protein analysis. Statistical analysis for the immunofluorescent staining was performed with an unpaired t-test. Statistical significance was set at *p* < 0.05. Values represent mean ± SEM

Deficient conversion of uterine spiral arteries may lead to a greater degree of intermittent perfusion of the intervillous space, which has been associated with common pregnancy complications, such as intrauterine growth restriction (IUGR) [26]. In order to translate the in vitro results to clinical manifestations of greater uteroplacental perfusion, we finally analyzed GLUT1 and GOT2 expression in term placental explants. For this purpose, we subjected term placental villi of cesarean section from healthy women to low flow rates (1 ml/min), as described previously for first trimester explants and to high flow rates (7.9 ml/min), to mimic the high FSS acting on IUGR placenta. We observed a downregulation of GOT2 protein in tissues exposed to high FSS (Fig. 8a and Fig. 8b). Next, we compared the expression of GOT2 in healthy control samples with IUGR placentas from three different cohorts. Quantification of immunofluorescent staining for placental GOT2, and immunoblotting (Fig. 8c, d and f) showed both a tendency to be decreased in IUGR compared to controls. qPCR analysis of two different cohorts showed a significant decrease of GOT2 mRNA expression (p = 0.0101) in IUGR compared to healthy control placentas (Fig. 8e and Additional file 1: Figure S8a, b). GLUT1 was not affected in IUGR pregnancies on protein level (Fig. 8f–h), which is in good agreement with available literature [13]. However, *HK2* and *PFKP* showed the tendency to be upregulated in IUGR placentas (Fig. 8i, j).

## Discussion

**Fig. 9:**
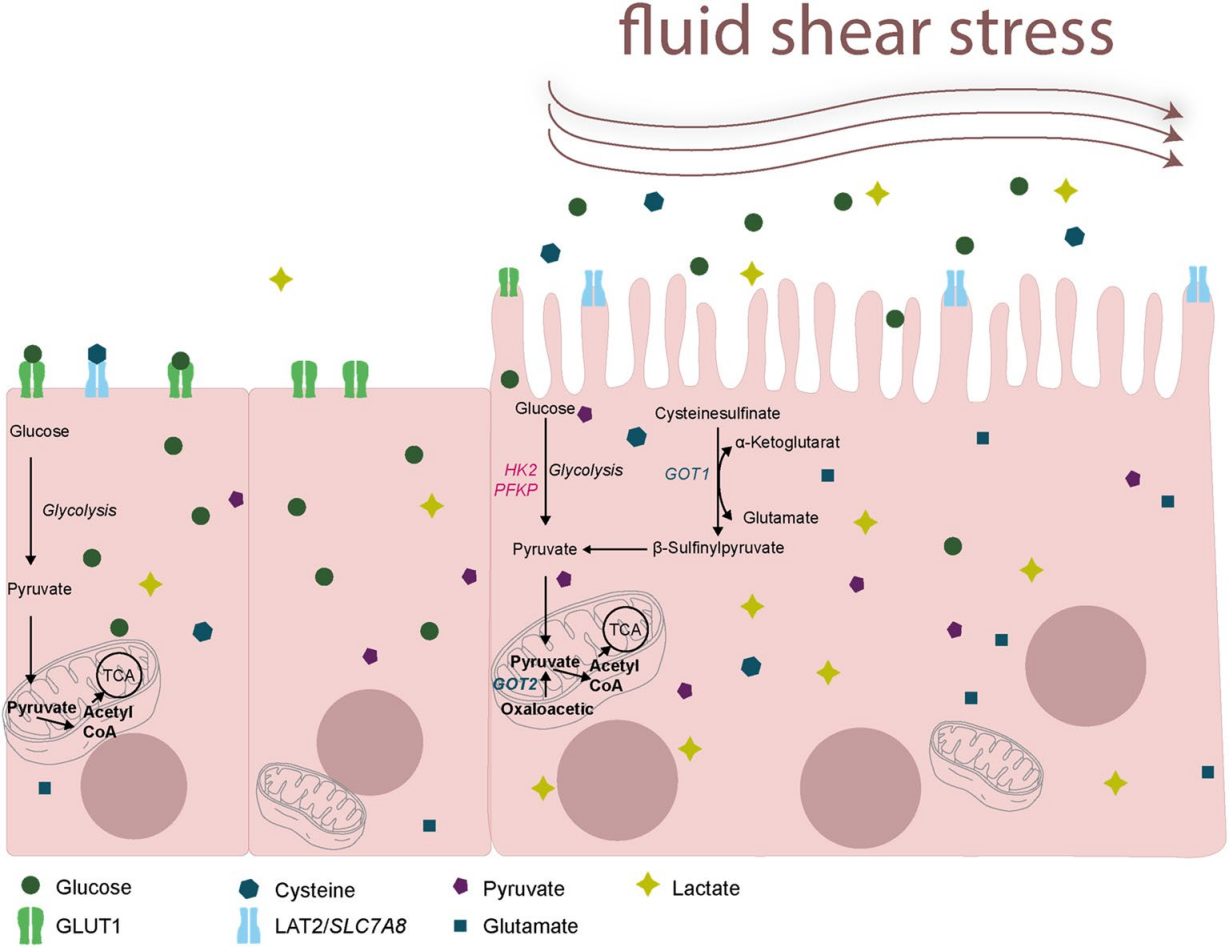
Summary of the metabolic pathway in placental cells. Summary of the metabolism in cells and tissue exposed to shear stress. The main glucose transporter GLUT1 is downregulated, and lower glucose levels in the cells and higher glucose levels in the supernatant were observed. Genes involved in the glycolytic pathway, like *HK2, PFKP* and *PGK1* are also downregulated in cells treated under flow conditions. Products of the glycolytic pathway, like pyruvate and the downstream product lactate are upregulated in cells and supernatant exposed to fluidic flow. A second pathway was observed, which could handle the energy maintenance in cause of low glucose consumption and downregulated glycolysis. Amino acid transporter *SLC7A8* is upregulated in cells treated under flow conditions, as well as the enzymes GOT1 and GOT2. Product from the conversion from cysteine to pyruvate is glutamic acid, which is upregulated in cells exposed to shear stress

Our current study demonstrates a downregulation of GLUT1, followed by a decrease of glycolysis in differentiated villous trophoblasts, as a consequence of FSS. Importantly, overall intracellular energy balance does not seem to be affected, as cells show no significant differences in pyruvate levels but higher lactate levels, when cultured under fluidic flow. Furthermore, our data suggest that induction of GOT enzymes contributes to an alternative pathway that may compensates the impaired glycolysis by conversion of amino acids into pyruvate (Fig. 9).

It should be noted that the placenta is an organ of limited life span, which, however, has to quickly expand in mass and adapt to maternal physiology as well as fetal demands at different stages of pregnancy. Thereby, the epithelial-like trophoblast covering developing villous trees is increasingly exposed to FSS. To date, only a few studies have focused on effects of FSS on the developing placenta in the first part of pregnancy [23]. Recent studies demonstrate effects of FSS on the trophoblast of an artificial placental barrier [12], and suggest that low fluidic flow has an impact on Ca^2+^ influx and cell differentiation, including microvilli formation [10, 12]. Additionally, FSS mediates an upregulation of glucose transporter GLUT1 with the full onset of fluidic flow [13] and in undifferentiated BeWo cells [10], which we confirmed with our fluidic system. Like the human placenta, many other adult organs in human, consisting of epithelial- and/or endothelial cells, are exposed to varying degrees of shear stress. Lung epithelial cells, for example, are exposed to airflow-related shear stress of approx. 0.5–3 dyne/cm^2^ that mediates upregulation of the TRPV receptors, a family of transient receptor potential cation channels (TRP channels) highly selective for Ca^2+^ [27]. Interestingly, the family of TRPV channels seems to be affected by shear stress in a broad panel of different cell types, including the syncytiotrophoblast of the placental barrier [10]. In particular, epithelial cells from organs that fulfill important resorption and/or exchange functions, such as intestinal enterocytes or renal epithelial cells (of the proximal convoluted tubule) depend on proper formation of microvilli on their apical surface. Renal epithelial cells show a higher Ca^2+^ influx in response to FSS (1 dyne/cm^2^) [28], whereas intestinal cells are exposed to low shear rates of up to 0.08 dyne/cm^2^ that promote formation of microvilli, as has been shown in colorectal adenocarcinoma cell line Caco-2 [29].

However, since the highly differentiated syncytiotrophoblast is the cell type exposed to maternal blood flow, we used forskolin-induced BeWo cells that underwent syncytialization before they were subjected to flow culture experiments. Exposure of differentiated BeWo cells to a physiological shear rate, revealed a substantial effect on the trophoblastic transcriptome, assessed with RNA-Seq and bioinformatics analyses. Beside genes belonging to the ribosomal pathway, genes involved in the energy metabolism, such as glycolysis, amino acid metabolism, and mitochondrial physiology, are affected in differentiated trophoblasts exposed to shear stress.

Main nutrients for the developing fetus and the placenta are glucose, amino acids and lipids, with glucose stored as glycogen as a glucose reserve in the placenta for the developing fetus [30]. With the downregulation of genes encoding for key enzymes of the glycolysis *HK2* and *PFKP* in the syncytiotrophoblast layer in response to moderate shear stress, we suggest that there is an effect on the glucose availability for the fetus, by increasing the net transport of glucose to the growing fetus [31]. Decrease in key enzymes of the glycolysis after onset of fully established blood flow, was already demonstrated by Prater et al. [32]. In their study they showed a lower glycolytic activity in second trimester placentas compared to first trimester placentas, mentioning that this is possible due to the different acidity or the onset of shear stress, which is in a good agreement with our results. Our data suggest that differentiated trophoblasts cultured under static conditions are highly glycolytic and rather do not use mitochondrial oxidative phosphorylation for ATP production: Mass spectrometry data of the metabolites of the glycolytic and TCA cycle support this notion. Metabolites resulting from the activity of key glycolytic enzymes were decreased in cells cultured under flow, like glucose-6-posphate and fructose-1,6-bisphosphate. Interestingly we observed an accumulation of phosphoenolpyruvate, which indicates downregulation of pyruvate kinase, the last enzyme of glycolysis. Therefore, we speculate that in trophoblasts exposed to shear stress, a shift in the metabolic state slightly away from glucose consumption and towards oxidative phosphorylation derived ATP production may occur. This is in a good agreement with previous studies, where they are showing onset of oxidative phosphorylation at the second trimester of pregnancy, after full establishment of utero-placental blood perfusion [32]. As the cells are highly glucose dependent regardless of cultured under static- or fluidic flow conditions, it is plausible that the cells undergo a shift from anaerobe to aerobe glycolysis. Mass spectrometry data showed a significant decrease of citrate/isocitrate, indicating high TCA activity. Slightly higher α-ketoglutarate levels and significantly higher fumarate levels further strengthen this interpretation.

Observed relative high lactate levels may be explained by increased cell cytotoxicity in response to FSS, but analysis of released LDH and cleaved caspase 3 did not indicate adverse effects on viability, which is in good agreement with a recent study showing better structural and biochemical integrity of placental tissue from flow than from static cultures [33]. Increased lactate levels may be the consequence of the so-called Warburg effect, in which cells generate energy predominantly by aerobic glycolysis, followed by lactate fermentation, rather than by the usual route via the TCA cycle. However, due to lower glycolytic activity and normal activity of the mitochondria this pathway may rather be excluded. Our RNA-Seq data indicate a shift from the glycolytic to the amino acid pathway. With GOT1 (cytosolic) and GOT2 (mitochondrial) we identified two enzymes that are able to support the glycolysis in context of energy maintenance, by converting cysteine to pyruvate under FSS. Of note, GOT-mediated conversion of cysteine to pyruvate is accompanied by the formation of sulfide and hydrogen sulfide (H_2_S) as byproducts [34, 35]. In the placenta, the activity of GOT increases during first trimester of pregnancy with a peak at the end of the first trimester, the window of time when perfusion of the intervillous space with maternal blood is fully established [36]. This is in good agreement with the findings of Abrego et al. who showed that epithelial cells can use the metabolism of transaminase enzymes for energy production [37], suggesting that GOT enzymes may support the glycolysis by energy production. Moreover, induction of GOT can be important for angiogenesis and consequently the formation of new blood vessels [38]. Our data suggest that GOT2 is upregulated in CTs and the SCT from first trimester placental explants in response to FSS, with a significant upregulation in the SCT layer, which is very likely due to its spatial location, as it is directly affected by fluidic flow. The upregulation of GOT2, but not GOT1 in the SCT layer implicates that upregulation of both variants is not necessary. However, with silencing of GLUT1 transporter in syncytialized BeWo cells we were able to identify a link between glucose availability and the expression of GOT1, whereas GOT2 was not affected. As glucose can diffuse from maternal circulation into the syncytiotrophoblast independent of GLUT1, just following the glucose gradient, we additionally reduced the glucose availability for the cells, which led to an upregulation of the GOT2 enzyme.

We can only speculate about the discrepancy that downregulation of GLUT1 led to upregulation of GOT1 in BeWo cells, whereas it was associated with increased GOT2 levels in first-trimester placental explants. One crucial aspect is the model itself, as BeWo were exposed as a monolayer cell culture to fluid flow, without underlying additional cells. In contrast, the explant model comprises several different cell populations, and in particular the mononucleated CT layer underneath the SCT can be assumed to participate in glucose consumption and thereby makes comparison with the cell line model difficult. Another point that complicates comparison of the cell model with the placental explants is the differential expression pattern of the GOT variants in the villous trophoblast compartment. Since GOT1 is predominantly expressed in the CT layer of the first trimester placenta and therefore not directly exposed to fluid flow, only indirect effects mediated through the SCT can be assumed.

As GOT2 is significantly dysregulated in the SCT, which is in direct contact with fluidic flow, we investigated this enzyme in placentas from pregnancies complicated with IUGR, a pregnancy pathology associated with abnormal placentation rooting in early stages of pregnancy. It has become increasingly acknowledged that IUGR is associated with impaired transfer of glucose, amino acids and lipids through the placental barrier to the developing fetus [14, 39, 40].

We observed no change in GLUT1 expression in pregnancies affected by IUGR, which is in good agreement with previous studies [13, 14, 41]. The fact, that the placental expression of the glycolytic key enzymes was upregulated, while GOT2 expression was downregulated in IUGR cases, may indicate that the placental tissue uses much of the glucose for its own energy production, resulting in reduced transplacental glucose transfer. In this regard, further studies examining the function of GOT2 by knockdown or overexpression in a placental barrier model that considers multiple cell layers are warranted. However, it should also be stressed at this point that our in vitro models are suitable to demonstrate short-term effects of fluid flow on the villous trophoblast, whereas in the etiology of IUGR, long-term mechanisms may be involved, usually occurring several weeks before the appearance of clinical signs of the pathology.

Moreover, uteroplacental shear stress has an impact on this placenta-associated pregnancy pathology. At the beginning of pregnancy, EVTs originating from the placental villous trees invade the lumen of maternal spiral arteries. This invasion contributes to substantial remodeling of the maternal spiral arteries from tightly coiled vessels (in the non-pregnant state), to wide-bore, low-resistance conduits that open into the intervillous space and enable uteroplacental perfusion with low shear stress acting on the placenta [7, 26]. However, in IUGR cases, insufficient spiral artery remodeling may result in turbulent blood flow, including high-velocity jets and vortices in the intervillous space with elevated wall shear stress at the villous surface. This assumption is based on recent in silico approaches, such as by Roth et al. [42], who focused on inflow regions of a spiral artery, including a substantial spiralized segment of the artery and the first 2 mm of the intervillous space and the villous tree proximal to the opening of the spiral artery. Of note, this region was three-dimensionally reconstructed from serial histological sections. Exemplary Doppler flow data of uterine arteries from a clinically normal patient and from patients with IUGR were then used to feed the simulations throughout three full maternal heart cycles. The calculations found elevated wall shear stress levels at the surfaces of the villous trees in IUGR. There is evidence, that hypertension may lead to increased FSS on the placental SCT and subsequently to IUGR. This was suggested by the above mentioned in silico approach by Roth et al. showing high-velocity jets in the entry region into the proximal IVS and peak velocities that were higher by a factor of 5 in IUGR and a factor of 4 in IUGR combined with preeclampsia (IUGR/PE) compared to control. The calculated levels of wall shear stress at the surfaces of the villous trees were elevated in both pathological conditions, but were slightly more pronounced in IUGR than in IUGR/PE. In addition to EVT-mediated remodeling of spiral arteries, estrogen, H_2_S and nitric oxide play important roles in pregnancy-associated uterine vasodilation [35]. Lower H_2_S levels have been described for pathological placentas, including cases affected by IUGR [43]. Interestingly, both GOT1 and GOT2 are altered in such placenta pathologies, resulting in less H_2_S being provided via this pathway. Considering that the CT population declines with increasing duration of pregnancy, GOT1 may not play such a major role in this complication.

Our data were mostly generated in vitro under cell- and tissue culture conditions, which do not fully reflect the in vivo situation. A limitation of our current flow culture model is that the system does not account for circulating maternal immune cells, which could be activated by FSS to release pro-inflammatory cytokines into the intervillous space, negatively contributing to IUGR. Moreover, the placenta can be considered as a very dynamic organ that might quickly adapt to changes in fluidic flow. For example, this is the case when maternal blood turbulences in uteroplacental perfusion trigger activation of maternal platelets on the villous surface, which consequently leads to perivillous fibrin deposition. The latter indirectly contributes to shaping of placental villi and the intervillous space [44, 45]. Finally, it should be mentioned that different shear rates may act on different areas of the placental surface [8], but this will be the subject of follow-up studies.

## Conclusion

Our study suggests that onset of uteroplacental blood flow is accompanied by a shift from a predominant glycolytic- to an alternative amino acid converting metabolism in the syncytiotrophoblast. This shift could represent a supporting energy pathway for the syncytiotrophoblast itself, allowing increasing net transport of maternal glucose across the placental barrier to the growing fetus. Rheological alterations in the intervillous space with exaggerated FSS at the villous surface, as has been suggested for IUGR [41], may disturb this alternative amino acid pathway in the syncytiotrophoblast and could contribute to this pregnancy pathology.

## Methods

### Ethics statement

The study was approved by the ethical committee of the Medical University of Graz (31-019 ex 18/19). Placental villous tissue was obtained from electively terminated pregnancies with informed consent of healthy women. Exclusion criteria were a maternal age under 18, maternal BMI > 25, and maternal pathologies.

### Sample collection

Samples from different patient cohorts were used for validation. First trimester placenta tissues were obtained between 6 and 10 weeks of gestation from women undergoing legal elective pregnancy terminations. Term placental tissue was collected from caesarean section of healthy patients in Graz, Austria (gestational age 32–38 weeks n = 10). Healthy third-trimester and IUGR samples were collected at the University Hospital Graz, Austria (Additional file 1: Table S2). Villous samples were washed and snap frozen in liquid nitrogen for later use for RNA and protein analyses. Paraffin blocks of control and IUGR samples were kindly provided from the Department of Anatomy II, LMU Munich, Germany for immunofluorescent staining (Additional file 1: Tab. S3). IUGR was clinically diagnosed if the growth parameters of the fetus determined by ultrasound (e.g. femur length, abdomen and head circumference) were above the 10th growth percentile during the first two trimesters and then dropped below the 10th growth percentile. The obstetricians made this diagnosis during routine clinical monitoring. Additional IUGR and controls were sampled by the Charité in Berlin, Germany [46] (Additional file 1: Table. S4) to expand the cohort from Graz for qPCR analysis. All participating women gave a written informed consent, and all studies were approved by the local ethics committee.

### Fluidic flow culture

Trophoblast cell line BeWo (European Collection of Cell Cultures, ECACC, Salisbury, UK) was seeded in 12-well plates containing glass plates (Ø12 mm, Roth) in a density of 200.000 cells per well. Next day, cells were differentiated with 20 µM forskolin (TOCRIS bioscience) for 48 h. Thereafter, glass plates were transferred to flow chambers. For cultivation DMDM/F12 (1:1) (Gibco; Cat.Nr.:2133–020) supplemented with 10% (v/v) fetal bovine serum (FBS, HyCloneTM, Cat.Nr.:SV30160.03; heat inactivated at 56 ℃ for 1 h), 1% (v/v) l- Glutamine and 0.1 U/ml Penicillin (Gibco, Cat.Nr.: 15,140–122, 0.1 µg/ml Streptamycin) was used. For different glucose concentrations, DMEM low glucose (11885084) was used and either mixed 1:1 with no glucose medium (Gibco, Cat.Nr.: 11966025). For 2.5 g/l glucose concentration no glucose medium was mixed 1:1 with high glucose Medium (Gibco, Cat.Nr.: 10566016). Flow chambers (QV500, Kirkstall, North Yorkshire,UK) were connected to a tubing system (Kirkstall; 2 × 1/16″ 7 diameter 22 cm length, 1 × 3/32″ diameter 22 cm length) and a 30 ml reservoir bottle. The flow cycle was placed in a TEB500 flow bioreactor (Ebers Medical Technology SL, Zaragoza, Spain). Each flow cycle included three chambers and 18 ml culture medium. The perfusion rate was 3 ml/min, equaling to 0.95 dyne/cm^2^. Calculation of dyne/cm^2^ is provided in Supplementary materials. In parallel, cells grown on glass plates as described above were transferred to 6-well dishes, including 6 ml culture medium, and served as a control. The bioreactor was adjusted to 2.5% O_2_ and 5% CO_2_ concentration with a temperature of 37 ℃.

Placental villous tissue (n = 4; 8.6 ± 1.6 weeks of gestation) was processed within 1–4 h after elective surgical termination of pregnancy. Placental tissue was washed in PBS, dissected and mounted on metal plates as previously described [33]. Metal plates were transferred to flow chambers as described for BeWo cells, villi in 6-well plates served as a control. The flow rate and microenvironment was adjusted as described for cell culture. For the cultivation DMEM (including low glucose, pyruvate, l-glutamine) supplemented with 10% FCS (HyCloneTM; Gibco) and penicillin/streptomycin (Gibco) was used. Placental term explants were processed within one hour after cesarean section of healthy control patients. Tissue was processed as previously described for first trimester tissue, for the flow culture, flow rates of 1 ml/min and 7.9 ml/min were used.

### LDH assay

For LDH Assay, supernatants from static and flow culture was collected and centrifuged at 1500^x^g for 10 min at 4 ℃. The LDH Assay (LDH Cytotoxicity Detection Kit, Takara, Japan) was performed according to the manufacture’s protocol, and samples were measured in duplicates.

### hCG assay

The hCG content in the culture supernatant was measured using routine immunoassays at the department of Obstetrics and Gynecology at the Medical University of Graz (Dimension Xpand; Dade Behring Inc., Deerfield, IL, USA). Supernatants were centrifuged for 10 min at 1500^×^*g* and 4 ℃. Samples were stored at – 80 ℃ and subjected in groups to analyses. Obtained values were normalized to total cell protein.

### SLC2A1 silencing

BeWo cells were seeded in 24-well plates at a cell density of 150.000 cells/well and incubated overnight. Afterwards, cells were differentiated with 20 µM forskolin for 48 h. Thereafter, cells were transfected with a predesigned siRNA targeting human *SLC2A1* (2.5 pM, Silencer siRNA, ThermoFisher Scientifc) or non-targeting negative control siRNA using Lipofectamine (Lipofectamine™ RNAiMAX Transfection Reagent, ThermoFisher Scientific) as a transfection reagent in 1 ml serum free culture medium for 24 h.

### RNA isolation and qPCR analysis

Total RNA of cells was isolated with RNA isolation kit (ReliaPrep RNA Cell Miniprep System, Promega) according to the manufacturer’s protocol. First trimester explants were homogenized with stainless steel beads (5 mm, Qiagen). Samples including beads were oscillated with 50 1/s for 1 min four times, in between the repetitions samples were placed 1 min on ice. Subsequently, samples were sonified for 30 s with 10 cycles. Total RNA was isolated from homogenized samples with SV total RNA Isolation Kit from Promega. RNA concentration and purity were measured with NanoDrop ND-1000 Spectrophotometer (ThermoFisher Scientific, Massachusetts, USA). cDNA was generated by reverse transcription of 100 ng-1 µg total RNA using High-Capacity cDNA Reverse Transcription Kit (Applied Biosystems, Foster City, CA, USA), according to the manufacturer’s manual. mRNA expression was evaluated using qPCR, performed with SYBR Green (Biozym, CityVienna, Austria) using a Bio-Rad CFX384 cycler and specific primer pairs (Additional file 1: Table. S5). Ct values and relative quantification of gene expression were automatically generated by Bio-Rad CFX Maestro Software (Bio-Rad Laboratories; Hercules, CA, USA) using the expression of TBP, HPRT1 and B2M as reference, according to our RNA-Seq data, showing their stable expression across different culture conditions.

### RNA-Seq

RNA quality, three per condition, was determined using an Agilent 2100 Bioanalyzer (Agilent Technologies, Santa Clara, CA, USA); with RIN > 7. Sample processing was carried out at the Division Core Facility for Molecular Biology at the Centre of Medical Research at the Medical University of Graz. Briefly, ribosomal-depletion was performed with NEBnext rRNA Depletion Kit V2 (New England Biolabs; Ipswich, MA, USA; Cat.No. E7405) and the library was prepared with NEBnext Ultra II (non directional) RNA Library Prep Kit for Illumina (New England Biolabs; Ipswich, MA, USA; Cat.No. E7770). Index were used from NEBNext Multiplex Oligos, Set 1 (New England Biolabs; Ipswich, MA, USA; Cat.No. E7335S). The RNA-Seq was performed at Vienna BioCenter Core Facilities GmbH, samples were indexed and sequenced on an Illumina NovaSeq SP SR100 XP (single lane). The generated RNA-Seq raw data are accessible through NCBI Short Read Archive under the accession number PRJNA904961.

### Bioinformatics analysis

For differential gene expression analysis, all FASTQ files (three samples per condition) were analyzed using a RNA-Seq workflow created in Galaxy [47]. Quality of the sequence reads was checked with FastQC (*version 0.72*+galaxy1). Adapter sequences were removed with Cutadapt (Version 1.16.5), followed by a quality control with FastQC. Sequences were mapped to the human genome GRCh38.p13 with STAR 2-pass (Version 2.7.7a). Quality assessment of the alignment was performed with QualiMap (Version 2.2.2d+galaxy1) and MultiQC (Version 1.7). Gene counts of each sample were generated with htseq (Version 0.9.1). Differential gene expression analysis (DGEA) was conducted in R (Version 4.0.5) [48] using DESeq2 (Version 1.30.1) [49]. Genes with an absolute log2 fold change of greater or equal to log2(1.5) and an adjusted p-value of smaller or equal to 0.05 were considered to be significantly differentially expressed. The volcano plot and the heatmap were generated using the R packages EnhancedVolcano (Version 1.16.0) [50] as well as pheatmap (Version 1.0.12) [51]. GSEA was conducted using the R packages DOSE (Version 3.16.0) [52] and clusterProfiler (Version 3.18.1) [53]. An adjusted p-value equal or lower than 0.01 was used as a significance threshold. Results are presented for 3 ml/min vs static.

### Protein isolation and immunoblotting

Cells were washed with PBS and lysed in RIPA buffer (Sigma-Aldrich, Saint Louis, MO, USA) including protease inhibitor cocktail (Roche Diagnostics; Mannheim, Germany) and PhosSTOP EASYpack (Roche, Diagnostics; Mannheim, Germany). Placental explants were homogenized in RIPA buffer using stainless steel beads (5 mm, Qiagen). Samples including beads were four times oscillated with 30 1/s for 1 min and 1 min placed on ice between the oscillation steps. Subsequently, samples were sonified 10 cycles for 30 s. Cell- and tissue lysates were centrifuged at 8000 × *g* with 4 ℃ for 10 min. The total protein concentration was determined in clear supernatant according to the Lowry method. 20 µg total protein were separated on 10% Bis–Tris gels (NuPAGE, Novex, Life Technologies) and blotted on 0.45 µM nitrocellulose membranes (Hybond, Amersham Biosciences, GE Healthcare Life Sciences, Little Chalfont, UK). Blotting efficiency was validated by Ponceau staining (Ponceau S solution, Sigma Aldrich). Antibodies were tested on a whole membrane with BeWo and first trimester placental lysates. For experiments, membranes were cut in horizontal strips at expected molecular weight ranges for target proteins. Primary antibodies were incubated on membrane strips (Additional file 1: Table S6). Secondary antibodies were incubated on membranes for 2 h at RT and detection was performed using a chemiluminescent immunodetection kit (Western Bright chemiluminescence Substrate Quantus, Biozym, Austria). Images were acquired with WesternBright Quantum (Biozym, ref.no. 541015). Band densitometry was performed using Image Studio Lite 5.2 quantification software (Li-Cor Biosciences, Germany). Results are presented as a ratio of band densities of the target protein and reference proteins peptidylprolyl isomerase (PPIB, also referred to as cyclophilin B) or vinculin (VCL), with control samples set to one. Uncropped representative Western blot images, as acquired with the iBright^™^ software, are shown in Additional file 1: Figure S9.

### Immunofluorescence

Placental villi were fixed in paraformaldehyde (PFA; Diapath S.P.A., Martinengo BG, Italy) for 12–16 h and thereafter rinsed in PBS. After the dehydration step, samples were embedded in paraffin. Placenta sections were deparaffinized according to standard protocols and an antibody specific antigen retrieval was performed. Sections were cooled down and subsequently incubated for 45 min at RT with primary antibodies (Additional file 1: Table. S7). Secondary Alexa Fluor conjugated goat anti-mouse or anti-rabbit antibodies were incubated for 30 min at RT. Nuclei were stained with DAPI, and sections were mounted with ProLong^™^ Gold Antifade Reagent (Thermo Fisher Scientific). Imaging was performed with Olympus SLIDEVIEW VS200.

### Immunofluorescent analysis

Image analysis was performed on the whole-slide images using the image analysis software Visiopharm, version 2021.09.

*AI based analysis of glutamic oxaloacetic transaminase GOT:* Using Visiopharm software version 2021.09 we designed apps to assess GOT positivity on the SCT and CT layers. Therefore, five villi were preselected manually and a trained AI app detected the detailed villous regions. On this villous area the region representing the SCT and CT layer were detected using an AI trained app that separates villi stroma from the trophoplast layer based on the Cy5 stain, the AI app further separated the trophoblast layer into SCT and CT areas. In order to extract GOT signal peaks in Cy5 we subtracted a mean of Cy3 from the original Cy3 channel. GOT positive areas were assessed on the SCT and CT areas using an intensity threshold of 300 (on a 16-bit scale) on that Cy3 signal peak feature. GOT positive areas below a threshold of 6000 (on a 16-bit scale) and smaller than 3 µM^2^ as well as GOT positive areas smaller than 10 µM^2^ with an intensity lower than 7000 on more than 50% of the positive Area were excluded. The GOT positive areas were then reduced to a centerline in order to assess an approximate fraction of positive SCT and CT layer perimeter. *Manually assisted analysis of GLUT1 and GOT:* For the manually assisted analysis five villi regions were selected. On this villous area the region representing the SCT and CT layer were selected manually based on the CK7(Cy3) stain. In order to extract GLUT1signal peaks in Cy5 we subtracted a mean of Cy5 from the original Cy5 channel. GLUT1positive areas were assessed on the SCT and CT areas using an intensity threshold of 1200 (on a 16-bit scale) on that Cy5 signal peak feature. GLUT1positive areas below a threshold of 6000 (on a 16-bit scale) and smaller than 3 µM^2^ as well as GLUT1positive areas smaller than 10 µM^2^ with an intensity lower than 7000 on more than 50% of the positive Area were excluded. The GLUT1 positive areas were then reduced to a centerline in order to assess an approximate fraction of positive SCT and CT layer perimeter. The regions for GOT detection were selected equal to the analysis of GLUT1. In order to extract GOT signal peaks in Cy5 we subtracted a mean of Cy5 from the original Cy5 channel. GOT positive areas were assessed on the SCT and CT areas using an intensity threshold of 60 (on a 16-bit scale) in this Cy5 signal peak feature. GOT positive areas below a threshold of 160 (on a 16-bit scale) and smaller than 3 µM^2^ as well as GLUT1positive areas smaller than 10 µM^2^ with an intensity lower than 200 on more than 50% of the positive Area were excluded. The GLUT1positive areas were then reduced to a centerline to assess an approximate fraction of positive SCT and CT layer perimeter.

*IUGR:* Using Visiopharm software version 2021.09 we calculated mean intensities of GOT (Cy5) and GLUT1(Cy3) limited to the manually accessed trophoblast regions for five selected villi on each sample. Auto fluorescence of the sample was corrected by subtracting FITC intensities from the Cy5 channel.

### Mitochondrial ATP measurements

Prior to the measurements cells were transfected using the Transfection Reagent (Polyjet^™^; SL100688) purchased at SigmaGen Laboratories (Fredrick, MD, USA), to establish expression of mtAT1.03 [54]. Afterwards, cells underwent syncytialization for 24 h with Forskolin. For experiments, cells were perfused latest 1 h after the flow experiments with buffers containing physiological ion concentrations as follows in mM: 2 CaCl_2_, 138 NaCl, 5 KCl, 1 MgCl_2_, 1 HEPES, 10 d-glucose. In case of no Glucose containing buffer, nothing else was changed. pH was adjusted to 7.4 using NaOH. Imaging was done using an inverted microscope (Olympus IX73, Olympus, Vienna, Austria) equipped with an ApoN340 40 × oil immersion objective (Olympus), CCD QImaging Retiga R1 camera (Teledyne Photometrics, Tucson, AZ, USA) a LedHUB (Omnicorn Laserage Laserprodukte, Rodgau-Dudenhof, Germany) with a 455 nm LED combined with a CFP/YFP/RFP filter set (Olympus). For FRET measurements, the emission was separated in two halves of the camera using a dual-channel beam splitter DV2 (Teledyne Photometrics) and a CFP/YFP beamsplitter (505DCXR, Chroma Technologies, Bellows Falls, VT, USA). The microscope was coupled with a gravity-based perfusion system (PS-9D, NGFI, Graz, Austria; https://www.ngfi.eu) and a perfusion chamber (PC30; NGFI). The data were background-substracted using a background ROI and corrected for photobleaching using an exponential decay fit. Oligomycin was bought from Sigma Aldrich (Vienna, Austria) dissolved in DMSO [10 mM], stored at – 20 ℃.

### Detection of GOT1 and GOT2 mRNA with padlock probe approach

Padlock probe hybridization was used for the detection of GOT1, GOT2 and ACTB mRNA transcripts according to protocols previously described [55–57]. In brief, target mRNA is reverse transcribed, padlock probes were hybridized and ligated to the cDNA and amplified by rolling circle amplification. The rolling circle products are targeted by gene specific fluorescent labelled detection probes, resulting in bright fluorescent spot like signals [58]. Reference sequences were obtained from the National Centre for Biotechnology Information (NCBI) (GOT1: NM_002079.3 glutamic-oxaloacetic transaminase 1; GOT2: NM_002080.4 glutamic-oxaloacetic transaminase 2, and ACTB: NM_001101.5 actin beta). Design of the Padlock probes was performed with an open-source Python software package (https://github.com/Moldia/multi_padlock_design) as previously shown [59]. To increase specificity, each padlock probe backbone contains a common anchor sequence, allowing for dual-colour staining and reduction of false positive signals. To boost sensitivity of GOT1 and GOT2 detection, multiple reverse primers and multiple padlock probes where used. GOT1 and GOT2 expression were normalized to ACTB expression. Sequences of oligonucleotides can be found in Additional file 1: Tables S8–10. Quantification of in situ signals was performed using CellProfiler [60].

### Lactate and pyruvate measurements

Lactate was measured in culture supernatants and cell lysates using a fluorometric lactate detection assay kit (Sigma, Cat.no.: MAK064) with a detection sensitivity from 0.2 to 10 nmol according to the manufacturer’s protocol. Pyruvate was measured in cell lysates using a pyruvate assay Kit (Sigma, Cat.no.: MAK332) with a detection range between 0.2 and 50 µM, when applying the more sensitive fluorometric procedure according to the manufacture’s protocol. Fluorescence levels were measured with CLARIOstar^®^ plate reader from BMG Labtech.

### Glucose uptake

Cells grown on glass plates, as described above, were washed three times with PBS after flow culture and a 100 µl solution of the fluorescent glucose analogue 2-NBDG (30 µM; ThermoFisher Scientific) in DMEM without phenol red and glucose (A1443001, ThermoFisher Scientific) was incubated on cells for 1 h at 37 ℃. Supernatants were collected for fluorometric measurements and detected with CLARIOstar^®^ with an excitation wavelength of 465 nm and emission wavelength of 540 nm. Cells were washed again three times with PBS and subjected to fluorescent imaging with a Zeiss Observer.Z1. Mean intensity of intracellular 2-NBDG was analyzed with ZEN and normalized to counted nuclei.

### GOT activity assay

GOT activity was measured in cell lysates, harvested in buffer included in the kit, using a colorimetric AST/GOT Activity Assay Kit (Merck, Sigma-Aldrich) according to the manufacturer’s protocol. Measurements were done with Spark^®^ multimode microplate reader from TECAN.

### Mass spectrometry

After flow culture, cells were lysed in ice cold methanol.

Two-complementary HILIC chromatography based-methods were used to increase the metabolite coverage. For HILIC low-pH, cells were lysed in ice cold methanol. Proteins in cell lysates were precipitated by adding and vortexing (30 s) a 3:1 volume of ice-cold acetonitrile/methanol/acetone (1/1/1, v/v/v). After an additional precipitation step at 4 ℃ for 60 min, samples were centrifuged at 10,000 ×*g* for 10 min (Hereaus Biofuge pico, Hanau, Germany). The resultant supernatants were aspirated into clean Eppendorf tubes and evaporated under a gentle stream of nitrogen gas at room temperature. Dry extracts were resuspended in acetonitrile/water (1/1, v/v) to 200 µl sample volume and immediately stored at − 80 ℃ until further analysis. A full-scan mass-spectrometric measurement of each sample was achieved by a combination of Dionex Ultimate 3000 UHPLC (ultra-high performance liquid chromatography)—Q Exactive Focus mass spectrometer (Thermo Fisher Scientific, Waltham, MA, USA). Chromatographic separation was performed on an Acquity UPLC BEH Amide column (2.1 mm × 150 mm, 1.7 µM) (Waters Corporation, Milford, USA), thermostated to 40 ℃. Mobile phases additives for HILIC-low pH were ammonium acetate and 0.1% formic acid. The Q Exactive Focus mass spectrometer operated in MS^2^ discovery acquisition mode using a HESI II ion source. Full scan spectra from m/z 60 to 300 were acquired with a resolution of 70 000 (m/z 200) in positive mode. The following source parameters were used: Source voltage: 3.8 kV, source temperature: 300 ℃, sheath gas: 35 arbitrary units, aux gas: 10 arbitrary units, sweep gas: zero arbitrary units, capillary temperature: 300 ℃. Data were generated in a target lists as previously described [61], merged and used to process data with the Lipid Data Analyzer 2.8.2 (LDA) software tool. Data were normalized to protein content and set relative to static conditions.

For metabolomics high-pH, analyses were performed using a semiquantitative method following previous reports [62]. Briefly, media was removed from plates and cells were washed three times with ice-cold PBS. Next, 1 ml of extraction buffer (40% methanol, 40% acetonitrile, 20% water) was added carefully to each well and plates were then incubated at − 20 degrees for 20 min. Finally, samples were centrifuged at maximum speed and the supernatant was transferred to HPLC vials. Polar metabolites were analyzed using liquid chromatography couple to tandem mass spectrometry following the methodology described by Yuan et al. [63]***.*** We used an XBridge Amide column (100 mm × 2.1 mm × 4.6 µm, Waters, Manchester, UK), and separation was achieved with a Nexera UHPLC (SHIMADZU, Kyoto, Japan) with a gradient elution. Solvent additives for HILIC high-pH were ammonium acetate and 0.1% ammonium hydroxide. We used a SCIEX QTRAP 6500 triple quadrupole mass spectrometer (Applied Biosystems, Framingham, MA, USA) for detecting metabolites in both positive and negative polarity mode. Raw files were imported into Skyline software for peak integration, and we used the online tool MetaboAnalyst for further data normalization and statistical analyses. Metabolite peak areas were further normalized against the protein content per well using a Modified Lowry protocol.

### Statistical analysis

Statistical analysis was performed using GraphPad Prism 9.0.0. Outliers were identified with Grubbs’, α = 0.05, normal distribution of the samples was determined using Shapiro Wilk test. For analysis of BeWo cell experiments, statistical significance was analyzed using one sample t-test for data relative to static controls with a hypothetical mean, when data form static controls were set to 1. For analysis of patient cohort samples an unpaired t-test was used. For non-normal distributed samples, a Mann–Whitney-test was performed. p < 0.05 considered significance.

### Supplementary Information

**Additional file 1: Figure S1.** Validation of cell viability by staining of cleaved caspase 3. First trimester placental villi were stained for cleaved caspase 3 and E-cadherin. A positive control with first trimester villi treated with Staurosporin (2 µM) for 4 h as previously described [64] was used (a, arrowheads indicate caspase 3 positive cells). Placental villi cultured under flow (c) did not show increased caspase 3 activation, when compared to static conditions (b). **Figure S2.** Syncytiotrophoblast markers in response to fluidic flow. Gene expression patterns (relative rlog values) of differentiated BeWo cells treated either under static conditions (light grey legend) or flow culture (dark grey legend) for 24 h. **Figure S3.** Key steps in glycolysis. Glycolysis converts glucose to pyruvate (left pathway), while GOT1 and GOT2 convert cysteinesulfinate to pyruvate and taurine (centre path). Cysteine can also be converted to glutathione (right path). **Figure S4.** Effects of fluidic flow on the glucose uptake and their final processing in trophoblasts. Intracellular uptake of 2-NBDG-Glucose by differentiated BeWo cells either under static or fluidic flow culture (3 ml/min) for 24 h (a). Glucose concentration in supernatant in undifferentiated (DMSO) and differentiated (forskolin) BeWo cells, cultured under static or flow conditions (b). Expression of lactate dehydrogenase subunit B (c, encoded by *LDHB*) and component X of the pyruvate dehydrogenase (d, *PDHX*) was analyzed by qPCR. Scale bar in (a) represents 50 µm. Data are presented as mean ± SEM. Experiments were performed with a minimum of three different cell passages. Experiments with placental explants (c and d) were performed with four different placenta samples. **Figure S5.** Effect of fluidic flow culture on HUVEC. GLUT1 (encoded by *SLC2A1*) mRNA (a) and protein (b and c) expression, as well as glucose concentration in supernatant (d) and *HK2* mRNA expression (e) was analyzed in HUVEC, which were cultured for 24 h either under static or fluidic flow culture conditions. In the same samples, GOT1 mRNA (f) and protein (b, middle panel and g) as well as GOT2 mRNA (h) and protein levels (i and j) were analyzed. Data are presented as mean ± SEM. Static samples were used as controls and set to 1. Experiments were performed with five different cell passages. **Figure S6.** Flow-dependent change of intracellular amino acid composition and expression of enzymes involved in glutathione biosynthesis. Relative amount of amino acids (a) was determined by mass spectrometry in cell lysates from differentiated BeWo cells cultured for 24 h either under static conditions (white) or flow exposed cells (blue). Additional measured mass spectrometry data were able to detect intracellular (b) and extracellular (c) cysteine. Mass spectrometry data were normalized to total cell protein and static conditions set to 1. Glutathione (GSH) biosynthesis involves two ATP-dependent steps, including initial synthesis of γ-glutamylcysteine (γ-Glu-Cys) from l-glutamate and cysteine, catalysed by γ-glutamate-cysteine synthase (γ-GCS). In a second step, glycine (Gly) is added to the C-terminus of γglutamylcysteine by glutathione synthetase (GSS) (d). Expression of glutathione synthase (e, *GSS*) and glutamate-cysteine ligase catalytic subunit (f, *GCLC*). Data of amino acids are presented as mean ± SEM. Experiments were performed with five different cell passages; statistical analysis was done with one sample t-test. ns (not significant) Data from the glutathione pathway are presented as mean ± SEM and were obtained from three different experiments. Statistical analysis was done with an unpaired t-test for c and d. **Figure S7.** Effect of SLC2A1-silencing and different glucose availability on expression of HK2 and PFKP. *HK2* expression was determined by qPCR in differentiated BeWo cells after GLUT1-silencing (a), encoded by *SLC2A1*. Gene expression of *HK2* (b) and phosphofructokinase (c) encoded by *PFKP* in differentiated BeWo cells cultured under different glucose concentrations for 24 h. Data are presented as mean ± SEM. Experiments were performed with a minimum of three different cell passages. **Figure S8.** Placental GOT2 mRNA expression. *GOT2* expression was determined by qPCR in placental tissue cohorts from Berlin (a; controls, n=9 and IUGR, n=4) and Graz (b; controls, n=5 and IUGR, n=5). **Figure S9. **Uncropped representative Western blot images, as acquired with the iBright^TM^ software. **Table S1. **Differential gene expression between static vs 3 ml/min is provided online in a separate excel sheet (Supplemental Table 1_DGA). **Table S2.** Baseline characteristics of CTRL and IUGR cases (Cohort Graz, used for protein- and qPCR analysis). **Table S3.** Baseline characteristics of CTRL and IUGR cases (Cohort Munich, used for immunofluorescence). **Table S4. **Baseline characteristics of CTRL and IUGR cases (Cohort Berlin, used for qPCR). **Table S5**. Primer sequences. **Table S6.** Antibodies used for immunoblotting. **Table S7.** Primary antibodies for immunofluorescence staining. **Table S8.** Primers for in situ hybridization. **Table S9.** Padlock probes for in situ hybridization. **Table S10.** Detection Oligos in situ hybridization.

## Data Availability

The authors declare that all the relevant data are available within the paper and its Supplementary information file or from the corresponding author upon reasonable request. Correspondence and requests for materials should be addressed to M.G.
