# Supplementary material for: Fluid shear stress induces a shift from glycolytic to amino acid pathway in human trophoblasts
Source: Cell Biosci. 2023 Sep 8;13:163. doi: 10.1186/s13578-023-01114-3 (PMC10492287; doi:10.1186/s13578-023-01114-3)
Supplement: Supplementary file 1 — Additional file 1: Figure S1. Validation of cell viability by staining of cleaved caspase 3. First trimester placental villi were stained for cleaved caspase 3 and E-cadherin. A positive control with first trimester villi treated with Staurosporin (2 µM) for 4 h as previously described [64] was used (a, arrowheads indicate caspase 3 positive cells). Placental villi cultured under flow (c) did not show increased caspase 3 activation, when compared to static conditions (b). Figure S2. Syncytiotrophoblast markers in response to fluidic flow. Gene expression patterns (relative rlog values) of differentiated BeWo cells treated either under static conditions (light grey legend) or flow culture (dark grey legend) for 24 h. Figure S3. Key steps in glycolysis. Glycolysis converts glucose to pyruvate (left pathway), while GOT1 and GOT2 convert cysteinesulfinate to pyruvate and taurine (centre path). Cysteine can also be converted to glutathione (right path). Figure S4. Effects of fluidic flow on the glucose uptake and their final processing in trophoblasts. Intracellular uptake of 2-NBDG-Glucose by differentiated BeWo cells either under static or fluidic flow culture (3 ml/min) for 24 h (a). Glucose concentration in supernatant in undifferentiated (DMSO) and differentiated (forskolin) BeWo cells, cultured under static or flow conditions (b). Expression of lactate dehydrogenase subunit B (c, encoded by LDHB) and component X of the pyruvate dehydrogenase (d, PDHX) was analyzed by qPCR. Scale bar in (a) represents 50 µm. Data are presented as mean ± SEM. Experiments were performed with a minimum of three different cell passages. Experiments with placental explants (c and d) were performed with four different placenta samples. Figure S5. Effect of fluidic flow culture on HUVEC. GLUT1 (encoded by SLC2A1) mRNA (a) and protein (b and c) expression, as well as glucose concentration in supernatant (d) and HK2 mRNA expression (e) was analyzed in HUVEC, which were cultured for 24 h eith [file 13578_2023_1114_MOESM1_ESM.zip › CBIO-D-23-00070_R1_Supplementary Material.docx]

**Supplementary Materials**

**Fluid shear stress induces a shift from glycolytic to amino acid pathway in human trophoblasts**

Beatrice Anna Brugger^1^, Lena Neuper^1^, Jacqueline Guettler^1^, Désirée Forstner^1^, Stefan Wernitznig^1^, Daniel Kummer^1^, Freya Lyssy^1^, Julia Feichtinger^1^, Julian Krappinger^1^, Amin El-Heliebi^1,3^, Lilli Bonstingl^1,3^, Gerit Moser^1^, Giovanny Rodriguez-Blanco^4^, Olaf A. Bachkönig^5^, Benjamin Gottschalk^5^, Michael Gruber^1^, Olivia Nonn^1,6,7,8^, Florian Herse^6,7,8^, Stefan Verlohren^9^, Hans-Georg Frank^10^, Nirav Barapatre^10^, Cornelia Kampfer^10^, Herbert Fluhr^2^, Gernot Desoye^2^, Martin Gauster^1,^*

^1^Division of Cell Biology, Histology and Embryology, Gottfried Schatz Research Centre, Medical University of Graz, Graz, Austria

^2^Department of Obstetrics and Gynaecology, Medical University of Graz, Graz, Austria

^3^Center for Biomarker Research in Medicine (CBmed), Graz, Austria

^4^ Clinical Institute for Medical and Chemical Laboratory Diagnosis, Medical University of Graz, Graz, Austria

^5^Division of Molecular Biology and Biochemistry, Gottfried Schatz Research Centre, Medical University of Graz, Graz, Austria

^6^Experimental and Clinical Research Center, a cooperation between the Max‐Delbrück‐Center for Molecular Medicine in the Helmholtz Association and the Charité ‐ Universitätsmedizin Berlin, Berlin, Germany

^7^Max‐Delbrück‐Center for Molecular Medicine in the Helmholtz Association (MDC), Berlin, Germany

^8^Charité – Universitätsmedizin Berlin, corporate member of Freie Universität Berlin and Humboldt‐Universität zu Berlin, Berlin, Germany

^9^Department of Obstetrics and Gynaecology, Charité – Universitätsmedizin Berlin, corporate member of Freie Universität Berlin and Humboldt‐Universität zu Berlin, Berlin, Germany Clinic for Obstetrics, Charité Berlin, Berlin, Germany

^10^LMU Munich, Department of Anatomy II, Munich, Germany

*Correspondence:

Martin Gauster, https://orcid.org/0000-0003-0386-6857

Division of Cell Biology, Histology and Embryology,

Gottfried Schatz Research Center,

Medical University of Graz,

Neue Stiftingtalstraße 6, F/03/38,

8010 Graz, Austria

Tel: +43 316 385 71896

martin.gauster@medunigraz.at

**Methods**

Calculation of flow rates for dyne/cm^2^

𝜏 = 4 ∗ µ ∗ 𝑄 / (𝑟 ∗ 𝑝𝑖 ∗ h 2 ∗ 𝑡𝑢𝑏𝑒 𝑟𝑎𝑑𝑖𝑢𝑠)

𝜏 = dyne/cm²

µ = viscosity of the medium

Q = flow rate in ml/min

r = inner radius of the flow chamber

h = height of the chamber

**Tables**

**Supplementary Table 1**

Differential gene expression between static vs 3 ml/min is provided online in a separate excel sheet (Supplemental Table 1_DGA)

Supplementary Table 2

*Baseline characteristics of CTRL and IUGR cases (Cohort Graz, used for protein- and qPCR analysis)*

|  |  |  | *CTRL* | *IUGR* |
| --- | --- | --- | --- | --- |
| *n* |  |  | 5 | 5 |
| *Birthmode* | Prim.CS | n(%) | 80% | 100% |
| *Fetal sex* | m | n(%) | 40 | 40 |
|  | f | n(%) | 60 | 60 |
| *Fetal* | gestational age | days mean(SD) | 254,2 (9,26) | 249 (13,8) |
|  | placental weight | g mean(SD) | 468 (64,65) | 420 (72,93) |
|  | fetal weight | g mean(SD) | 2607,6 (294) | 1983 (549,63) |
|  | fetal length | cm mean(SD) | 48,36 (1,89) | 46,4 (2,07) |
|  | weight percentile | <3. Perc. (%) | 0 | 80 |
| *Doppler data* | A. umbilicalis | PI mean (SD) | 0.934 | 1,132 (0,37) |
|  | A. uterina left | PI mean (SD) | - | 1,038 (0,64) |
|  | A. uterina right | PI mean (SD) | - | 1,25 (0,67) |
| *Maternal* | BMI pregnancy | kg/m2mean(SD) | 28,24 (4,21) | 28.29 (4.61) |
|  | weight pregnancy | kg mean(SD) | 62,6 (5,77) | 64.4 (13.45) |
|  | weight at delivery | kg mean(SD) | 78,13 (8,25) | 74.02 (13.13) |

Supplementary Table 3

*Baseline characteristics of CTRL and IUGR cases (Cohort Munich, used for immunofluorescence)*

|  |  |  | CTRL | IUGR |
| --- | --- | --- | --- | --- |
| n |  |  | 18 | 20 |
| Birthmode | Prim.CS | n(%) | 83,33 | 80 |
| Fetal sex | m | n(%) | 44,44 | 55 |
|  | f | n(%) | 55,56 | 45 |
| Fetal | gestational age | days mean(SD) | 252,67 (25,66) | 234,7 (41,17) |
|  | placental weight | g mean(SD) | 417,50 (84,46) | 289,3 (99,16) |
|  | fetal weight | g mean(SD) | 2785,61 (718,63) | 1694,3 (1013,44) |
|  | fetal length | cm mean(SD) |  |  |
|  | weight percentile | mean (SD) |  |  |
| Doppler data | A. umbilicalis | PI mean (SD) |  |  |
|  | A. uterina left | PI mean (SD) |  |  |
|  | A. uterina right | PI mean (SD) |  |  |
| Maternal | BMI pregnancy | kg/m2mean(SD) |  |  |
|  | weight pregnancy | kg mean(SD) |  |  |
|  | weight at delivery | kg mean(SD) |  |  |

Supplementary Table 4

*Baseline characteristics of CTRL and IUGR cases (Cohort Berlin, used for qPCR)*

|  |  |  | CTRL | IUGR |
| --- | --- | --- | --- | --- |
| n |  |  | 9 | 4 |
| Birthmode | CS | n(%) | 88,89 | 50 |
| Fetal sex | m | n(%) | 66,67 | 50 |
|  | f | n(%) | 33,33 | 50 |
| Fetal | gestational age | days mean(SD) | 265,56 (14,50) | 272,25 (8,42) |
|  | placental weight | g mean(SD) | 648,33 (253,63) | 405 (73,26) |
|  | fetal weight | g mean(SD) | 3292,78 (701,55) | 2485 (237,42) |
|  | fetal length | cm mean(SD) | 50,33 (5,19) | 47,75 (0,96) |
|  | weight percentile | <3. Perc. (%) | 0 | 100 |
| Doppler data | A. umbilicalis | PI mean (SD) | 0,86 (0,14) | 1,27 (0,15) |
|  | A. uterina left | PI mean (SD) | - | - |
|  | A. uterina right | PI mean (SD) | - | - |
| Maternal | BMI pregnancy | kg/m2mean(SD) | 27,93 (5,51) | 31,94 (8,31) |
|  | weight pregnancy | kg mean(SD) | 63,62 (12,68) | 72,55 (9,51) |
|  | weight at delivery | kg mean(SD) | 77,44 (14,05) | 84,08 (13,75) |

**Supplementary Table 5**

Primer sequences

| Primer | Sequence (5’ -> 3’) | Product length (bp) |
| --- | --- | --- |
| *SLC2A1* forward | TGTCTTCTATTACTCCACGAGCA |  |
| *SLC2A1* reverse | CTCCACCACAAACAGCGAC | 121 |
| *HK2* forward | CATCTGCTTGCCTACTTCTTCAC |  |
| *HK2* reverse | ACTCTCCGTGTTCTGTCCCA | 99 |
| *PFKP* forward | CCCTTCGACATCAGGGATCTG |  |
| *PFKP* reverse | GCAGCTCTCATTTCTGAGCAC | 226 |
| *PGK1* forward | GGTCCTGAAAGCAGCAAGAAGT |  |
| *PGK1* reverse | GGCAGTGTCTCCACCACCTAT | 99 |
| *PDHX* forward | TGCTGACTCTGTAAAGGCTCT |  |
| *PDHX* reverse | TCGATGCCAAACATCCCCAA | 183 |
| *GOT1* forward | CTCAAGGAGAAGCGGGTAGGAG |  |
| *GOT1* reverse | AGCAGCGGAAAACACAGCATT | 168 |
| *GOT2* forward | CTTGAAGAGTGGCCGGTTTG |  |
| *GOT2* reverse | GGCAGAAAGACATCTCGGCT |  |
| *SLC7A8* forward | AGGCTGGAACTTTCTGAATTACG |  |
| *SLC7A8* reverse | ACATAAGCGACATTGGCAAAGA | 126 |
| *ACTB* forward | AAAGACCTGTACGCCAACAC |  |
| *ACTB* reverse | GTCATACTCCTGCTTGCTGAT | 219 |
| *TBP* forward | TGA CCC AGC ATC ACT GTT TC |  |
| *TBP* reverse | CCA GCA CAC TCT TCT CAG CA | 76 |
| *B2M* forward | GAT GAG TAT GCC TGC CGT GT |  |
| *B2M* reverse | TGT CTC GAT CCC ACT TAA CTA TCT | 70 |
| *HPRT1* forward | GAA AGG GTG TTT ATT CCT CAT |  |
| *HPRT1* reverse | CCA GCA GGT CAG CAA AGA ATT | 142 |

**Supplementary Table 6**

Antibodies used for immunoblotting

| Antibody | Company | Clon | Species | Dilution | Incubation time | Temp. |  |
| --- | --- | --- | --- | --- | --- | --- | --- |
| GLUT1 | Abcam | EPR3915 | mAb Rabbit | 1:10.000 | 1h | RT |  |
| GOT1 | Thermo Fisher | GT1255 | mAb Mouse | 1:10.000 | o/n | 4°C |  |
| GOT2 | Merck | 3E9 | mAb Mouse | 1:2.000 | o/n | 4°C |  |
| Vincullin | Invitrogen |  | pAb Rabbit | 1:1.000 | o/n | 4°C |  |
| Cyclophilin B | Cell Signaling | D1V5J | mAb Rabbit | 1:2.000 | o/n | 4°C |  |
| β-HCG | Thermo Fisher |  | mAB Rabbit | 1:50 | o/n | 4°C |  |

**Supplementary Table 7**

Primary antibodies for immunofluorescence staining

| Antibody | Company | Clon | Species | Dilution | AG retrival* |
| --- | --- | --- | --- | --- | --- |
| GLUT1 | Abcam | EPR3915 | mAb Rabbit | 1:500 | pH9 |
| GOT1 | Thermo Fisher | GT1255 | mAB Mouse | 1:500 | pH9 |
| GOT2 | Merck | 3E9 | mAB Mouse | 1:500 | pH9 |
| CK7 | Acris | RCK105 | mAb Rabbit | 1:500 | pH9 |
| CK7 | DB Biotech | RCK105 | mAb Mouse | 1:500 | pH9 |
| cleaved Caspase 3 | Abcam | 5A1E | mAB Rabbit | 1:200 | pH9 |
| E-Cadherine | Abcam | 4A2 | mAB Mouse | 1:200 | pH9 |

*Antigenretrival

**Supplementary Table 8**

Primers for in situ hybridization

| Primers | Sequences (5´ - 3´) | Primers | Sequences (5´ - 3´) |  |
| --- | --- | --- | --- | --- |
| ACTB_LNA | C+GG+GC+GG+CG+GATCGGCAAAG | **RV_GOT2_19** | GGTTGTCTCTGTTTCCT | |
| RV_GOT1_1 | TTGAAGACCAGGACAG | **RV_GOT2_20** | AGAGGCTGAAGACAGAA | |
| RV_GOT1_2 | GAAGTCGGCAGTGAG | **RV_GOT2_21** | TCTGTGTGAAGCTCT | |
| RV_GOT1_3 | GGCAAAACCCAGGGA | **RV_GOT2_22** | CCCAACTGGAGAAAC | |
| RV_GOT1_4 | CTCCACTTTCTTCACTAC | **RV_GOT2_23** | AACCGGGCAGAGACAA | |
| RV_GOT1_5 | GGTGTGTTCTTGTTGTTTGT | **RV_GOT2_24** | CAACAAAGGGAGGAGG | |
| RV_GOT1_6 | GGTGAGGACACATAGA | **RV_GOT2_25** | AGTGGGTGAAGCCTGAA | |
| RV_GOT1_7 | TCCAATCCTCTCTTCTC | **RV_GOT2_26** | AGATGGTGGTTCTTT | |
| RV_GOT1_8 | ATCATTCAGGAAGCCC |  |  |  |
| RV_GOT1_9 | GGTGCTTCATGACAG |  |  |  |
| RV_GOT1_10 | AAGGGGAACAGAAAC |  |  |  |
| RV_GOT1_11 | CACAGAAGAACTCGAA |  |  |  |
| RV_GOT1_12 | TTGGAGAAGGACTGG |  |  |  |
| RV_GOT1_13 | GTAGAGCCCGAAGTT |  |  |  |
| RV_GOT1_14 | CTCAGGTTCTTTTCCA |  |  |  |
| RV_GOT1_15 | AAAGGACTTGCAGGA |  |  |  |
| RV_GOT1_16 | GGGTTAGAGAGGGTG |  |  |  |
| RV_GOT1_17 | GTCCATTCCTCAAAGA |  |  |  |
| RV_GOT1_18 | TTTGATCAGTGATGTGG |  |  |  |
| RV_GOT1_19 | TGAAGCTGAACATGCC |  |  |  |
| RV_GOT1_20 | AAATTCGTCTCAAGGG |  |  |  |
| RV_GOT1_21 | CTCTAATCCCAGTCTC |  |  |  |
| RV_GOT1_22 | ATTTGCTTTGACCTCC |  |  |  |
| RV_GOT2_1 | GAATGGGATCTGGAGG |  |  |  |
| RV_GOT2_2 | AAAGGCTTCAGTGAC |  |  |  |
| RV_GOT2_3 | GCTATTGGTGTCCCTC |  |  |  |
| RV_GOT2_4 | CAGGTTGGTTTGGGC |  |  |  |
| RV_GOT2_5 | TGGGTGTGTGGTTTC |  |  |  |
| RV_GOT2_6 | GCCAGCATCCCTGAA |  |  |  |
| RV_GOT2_7 | AAATATCCTCCACAGC |  |  |  |
| RV_GOT2_8 | CACTCTGCTCTGGTATTTTT |  |  |  |
| RV_GOT2_9 | CAGGCATGCAGAAGAAG |  |  |  |
| RV_GOT2_10 | TTCCTTTTCTTCACCAC |  |  |  |
| RV_GOT2_11 | CAAAGAACGCAAAGAG |  |  |  |
| RV_GOT2_12 | CCTACACGCTCACCA |  |  |  |
| RV_GOT2_13 | TGCAGACCATAGTGAAG |  |  |  |
| RV_GOT2_14 | GCCATGACTTTCACT |  |  |  |
| RV_GOT2_15 | TGCCAATGATGCGGT |  |  |  |
| RV_GOT2_16 | TGAAACAGAACATGCC |  |  |  |
| RV_GOT2_17 | CGCTCCACCTGTTCA |  |  |  |
| RV_GOT2_18 | CGGCCATCTTTTGTCATGT |  |  |  |

+: the following base is LNA (locked nucleic acid) modified

Supplementary Table 9

*Padlock probes for in situ hybridization*

| Padlock probes | Sequences (5´ - 3´) |
| --- | --- |
| plp_GOT1_1 | TTCCGCAGGCCCAGC**CCTCAATGCTGCTGCTGTACTACTGCGTCTATTTAGTGGAGCC**TAGAGCATCTGACGCTACAGTCTTTGCCGAGG |
| plp_GOT1_2 | GGATGACTGCCATCC**CCTCAATGCTGCTGCTGTACTACTGCGTCTATTTAGTGGAGCC**TAGAGCATCTGACGCTAGGGAGCATATCGCAC |
| plp_GOT1_3 | CGTTGGTACAATGGA**CCTCAATGCTGCTGCTGTACTACTGCGTCTATTTAGTGGAGCC**TAGAGCATCTGACGCTAGCTGATTTCTTAGCG |
| plp_GOT1_4 | TACTGGGATGCAGAG**CCTCAATGCTGCTGCTGTACTACTGCGTCTATTTAGTGGAGCC**TAGAGCATCTGACGCTAATTCGGTCCTATCGC |
| plp_GOT1_5 | GGAGCAGTGGAAGCA**CCTCAATGCTGCTGCTGTACTACTGCGTCTATTTAGTGGAGCC**TAGAGCATCTGACGCTAGATTGACCCAACTCC |
| plp_GOT1_6 | TTGTGTCTGAAGGCT**CCTCAATGCTGCTGCTGTACTACTGCGTCTATTTAGTGGAGCC**TAGAGCATCTGACGCTAGGGCCATTCGCTATT |
| plp_GOT1_7 | TCTGACTGTGGTTGG**CCTCAATGCTGCTGCTGTACTACTGCGTCTATTTAGTGGAGCC**TAGAGCATCTGACGCTATGAGAGAGTCGGGAA |
| plp_GOT1_8 | ATTGTGGCCAGCACC**CCTCAATGCTGCTGCTGTACTACTGCGTCTATTTAGTGGAGCC**TAGAGCATCTGACGCTAGCCCAGGGAGCACGA |
| plp_GOT1_9 | CCTGGGACCTGGAAC**CCTCAATGCTGCTGCTGTACTACTGCGTCTATTTAGTGGAGCC**TAGAGCATCTGACGCTAGAAGCCCTCAAAACC |
| plp_GOT1_10 | CGGCCCCACATGAAG**CCTCAATGCTGCTGCTGTACTACTGCGTCTATTTAGTGGAGCC**TAGAGCATCTGACGCTAAGCCTCTGTTTAAAC |
| plp_GOT2_1 | ATGTGGAAATGGGAC**CAGTGAATGCGAGTCCGTCTTGCGTCTATTTAGTGGAGCC**TTGATATAGCGTATTCGCGGCTCCTGGTGGACCC |
| plp_GOT2_2 | TGTCTTTCTGCCCAA**CAGTGAATGCGAGTCCGTCTTGCGTCTATTTAGTGGAGCC**TTGATATAGCGTATTCGCGTAAGTTCAGCCGAGA |
| plp_GOT2_3 | GACTTCACAGGCGCT**CAGTGAATGCGAGTCCGTCTTGCGTCTATTTAGTGGAGCC**TTGATATAGCGTATTCGCGAAGACTTGCGGTTTT |
| plp_GOT2_4 | GTGGAAGGAAATAGC**CAGTGAATGCGAGTCCGTCTTGCGTCTATTTAGTGGAGCC**TTGATATAGCGTATTCGCGCCCGCGTCCGGAACA |
| plp_GOT2_5 | GCCAAGAACATGGGC**CAGTGAATGCGAGTCCGTCTTGCGTCTATTTAGTGGAGCC**TTGATATAGCGTATTCGCGCTCTGCCAATCATAT |
| plp_GOT2_6 | ACAATGGCTGCAAGA**CAGTGAATGCGAGTCCGTCTTGCGTCTATTTAGTGGAGCC**TTGATATAGCGTATTCGCGCCCAGATTTGCGAAA |
| plp_GOT2_7 | GACCAAATTGGCATG**CAGTGAATGCGAGTCCGTCTTGCGTCTATTTAGTGGAGCC**TTGATATAGCGTATTCGCGTGGCAACACATCACC |
| plp_GOT2_8 | TCCCTGGTGCGAGGA**CAGTGAATGCGAGTCCGTCTTGCGTCTATTTAGTGGAGCC**TTGATATAGCGTATTCGCGGGTCACCAAGTAATG |
| plp_GOT2_9 | GAATGATAGTGCAAG**CAGTGAATGCGAGTCCGTCTTGCGTCTATTTAGTGGAGCC**TTGATATAGCGTATTCGCGTCTCGGCATCCTCTT |
| plp_GOT2_10 | TTTGCGTGCTGCTAG**CAGTGAATGCGAGTCCGTCTTGCGTCTATTTAGTGGAGCC**TTGATATAGCGTATTCGCGGAGGATATTCTAGGC |
| plp_ACTB | AGCCTCGCCTTTGCC**TCTACGAGTTTGCAGTCACGTGCGTCTATTTAGTGGAGCC**GGTTGCTACGATGACTCACGCCCCGCGAGCACAG |

Padlock probes were 5’-phosphorylated. underlined: target complement sequence

**Supplementary Table 10**

*Detection Oligos in situ hybridization*

| Detection probes | Sequences (5´ - 3´) |
| --- | --- |
| D1_Atto425 | Atto425-CAGTGAATGCGAGTCCGTCT |
| D2_Atto488 | Atto488-TCTACGAGTTTGCAGTCACG |
| D3_TexasRed | TexasRed-CCTCAATGCTGCTGCTGTACTAC |
| D4_CY7 | CY7-TGCGTCTATTTAGTGGAGCC |

The detection probes were 5´conjugated with fluorophores (Atto425, Atto488, TexasRed, CY7 are fluorescent labels)

**Supplementary Figures**

**S**

**Supplementary Figure 1**

**
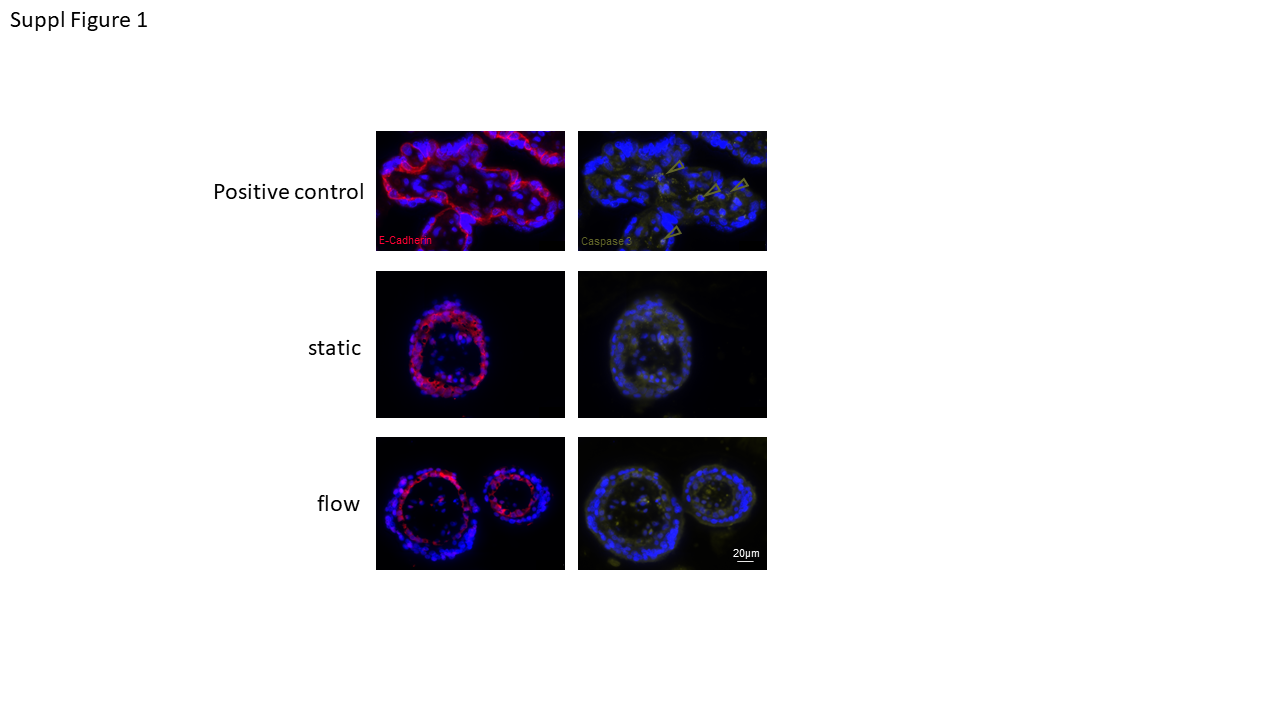
**

**Supplementary Figure 2**


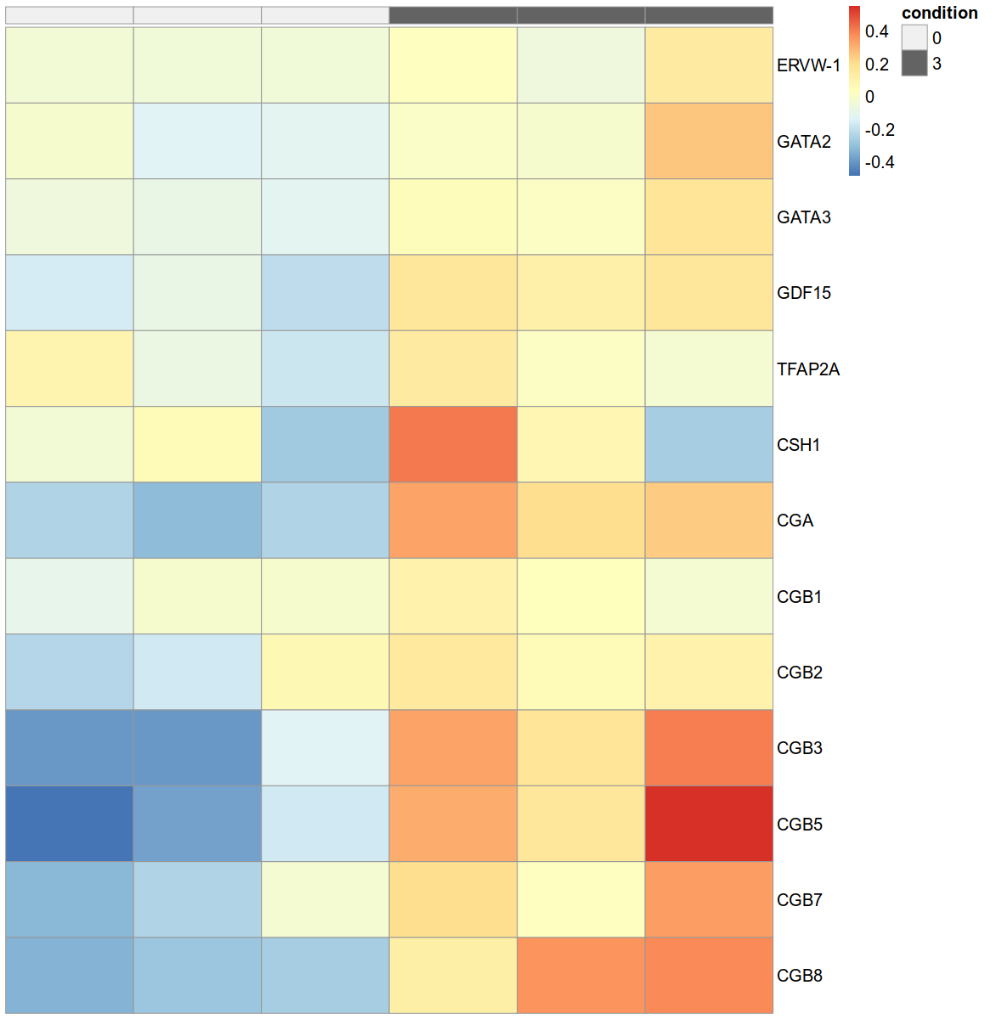


**Supplementary Figure 3**
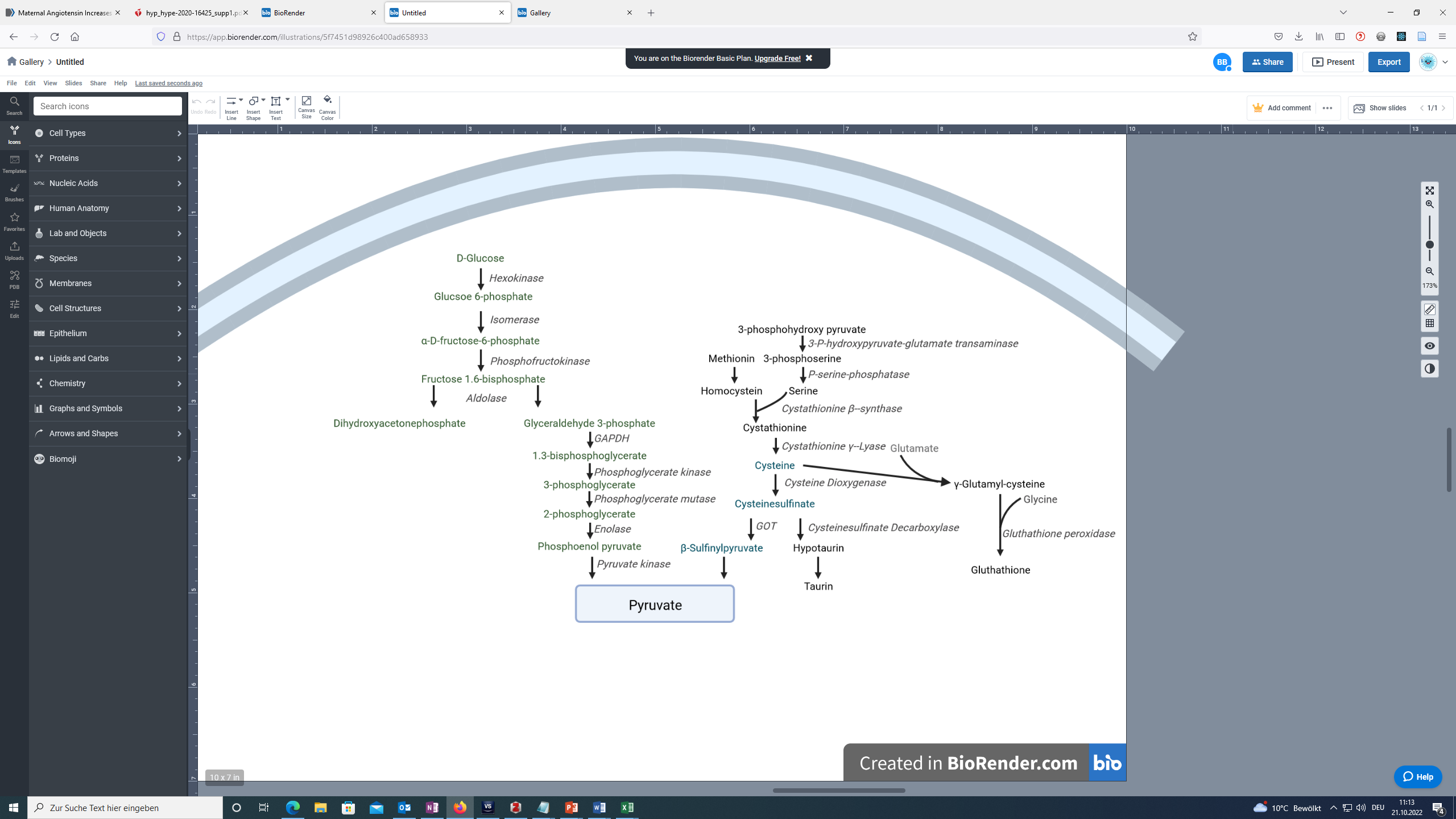


**Supplementary Figure 4**


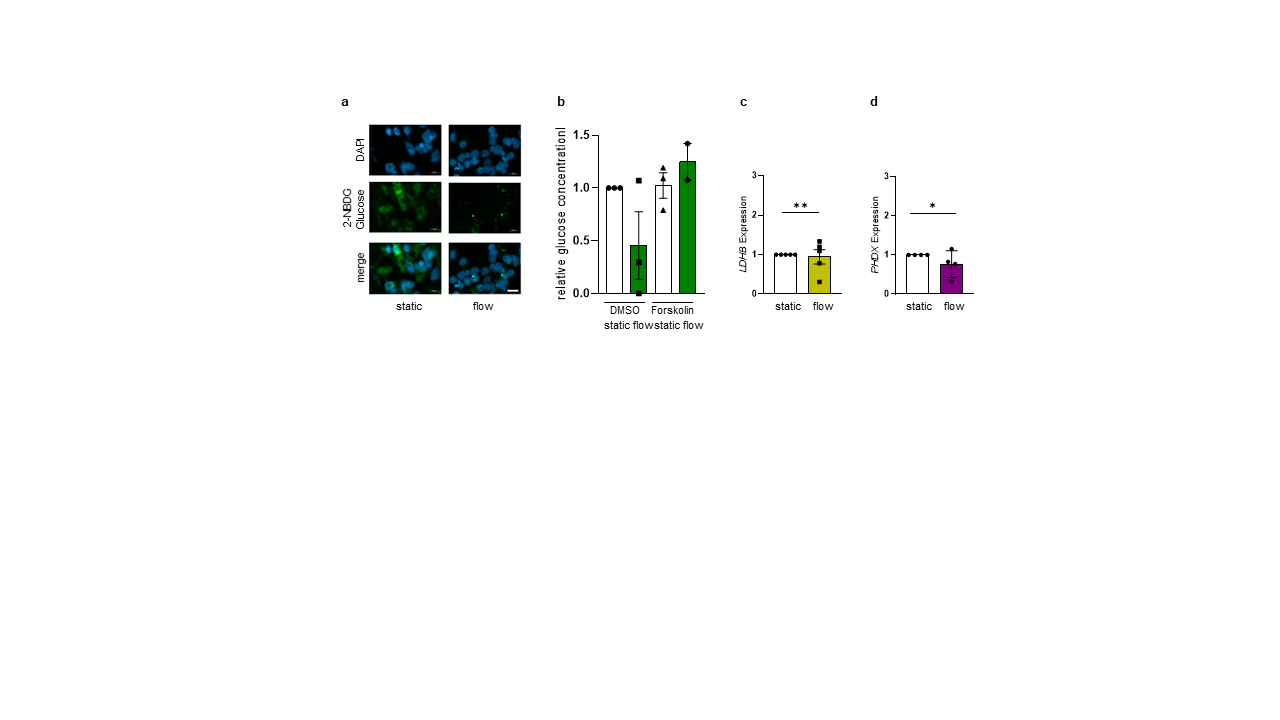


**Supplementary Figure 5**

**
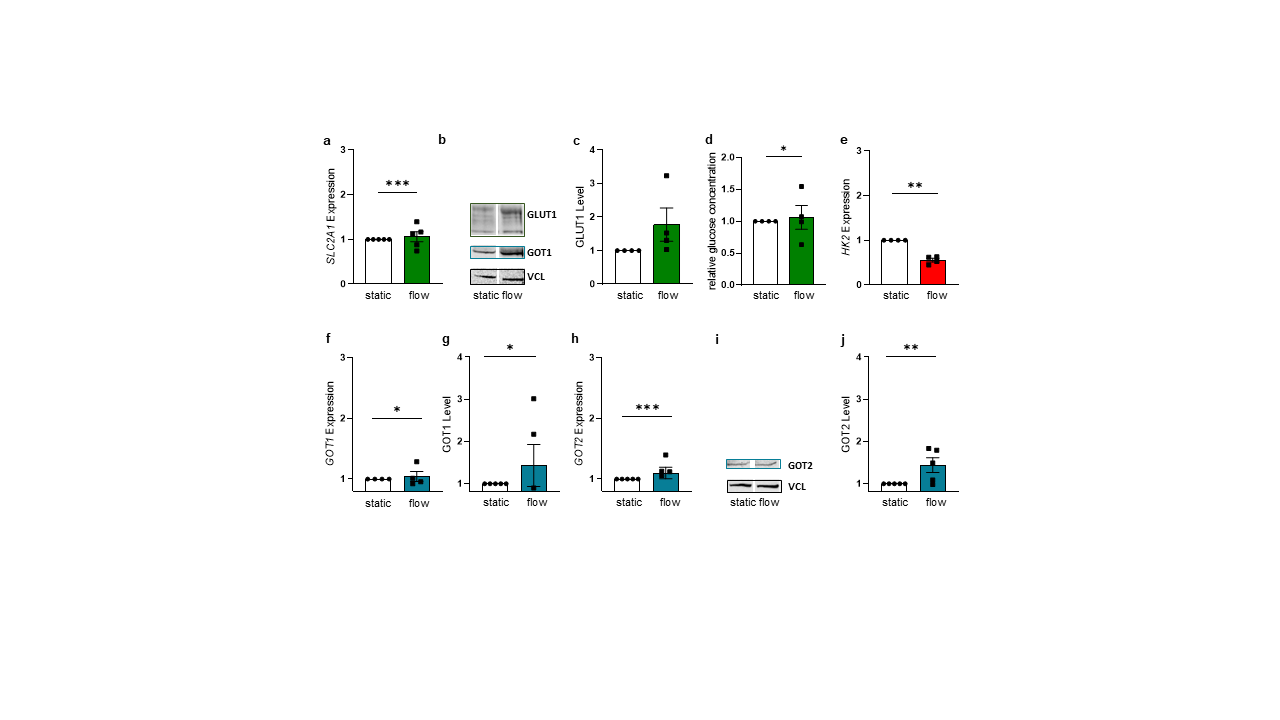
**

**Supplementary Figure 6**

**
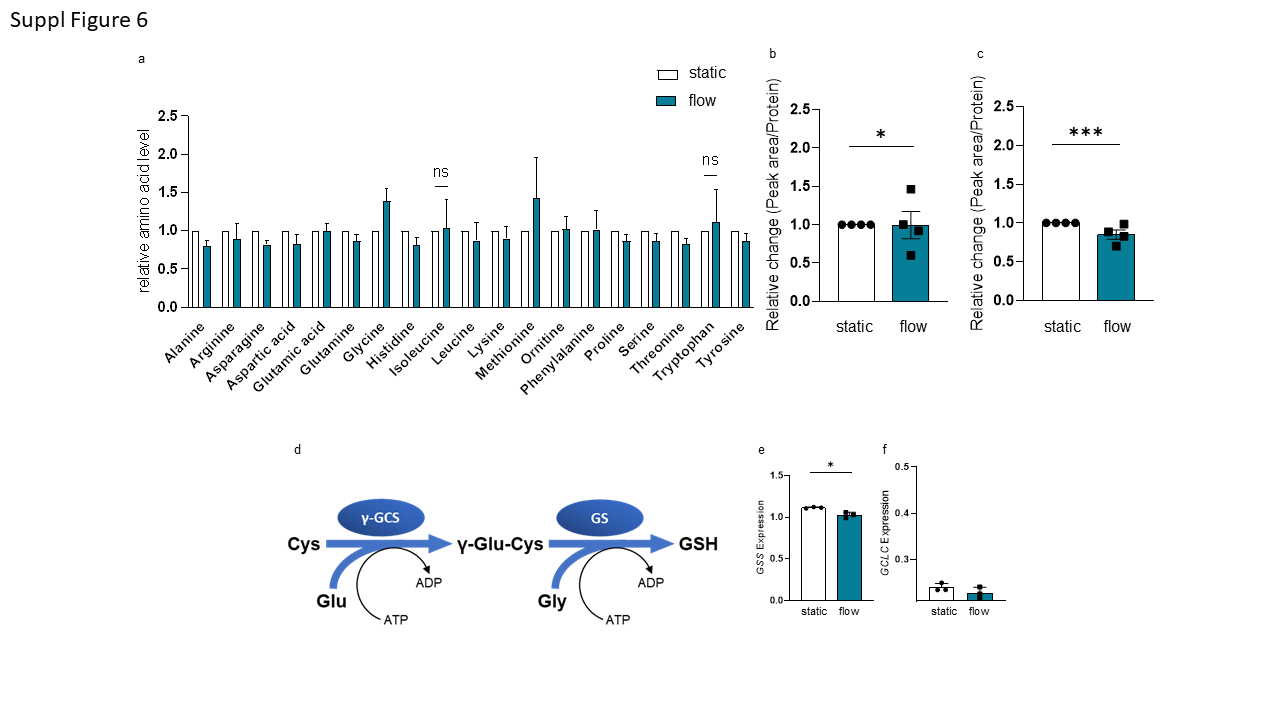
**

**Supplementary Figure 7**

**
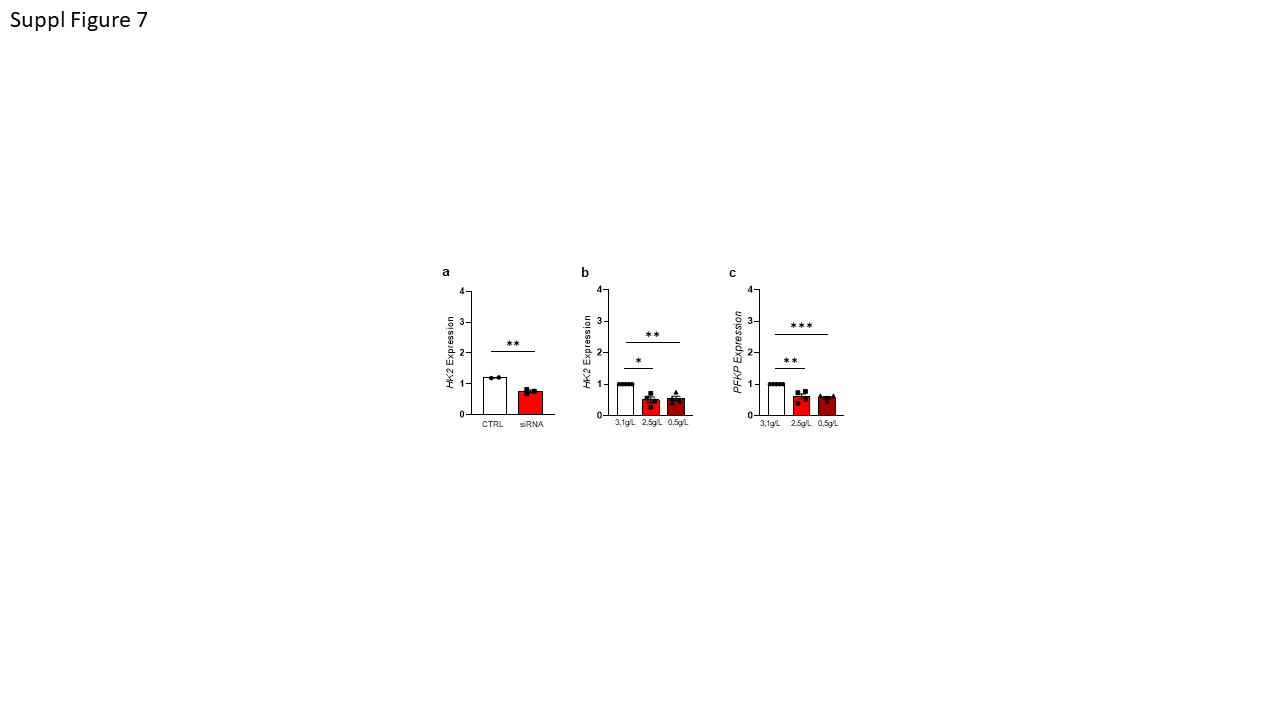
**

**Supplementary Figure 8**

**
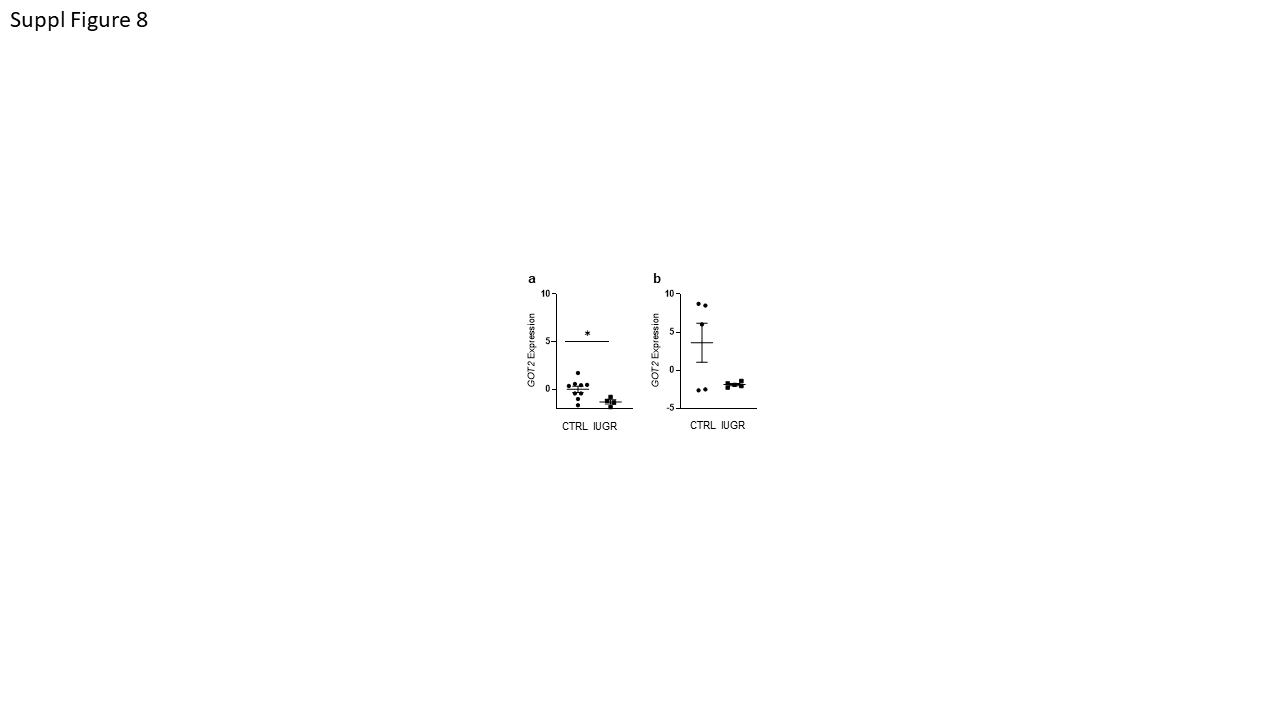
**
