# Supplementary material for: Fluid shear stress induces a shift from glycolytic to amino acid pathway in human trophoblasts
Source: Cell Biosci. 2023 Sep 8;13:163. doi: 10.1186/s13578-023-01114-3 (PMC10492287; doi:10.1186/s13578-023-01114-3)

Supplementary Figure 9

Uncropped representative Western blot images, as acquired with the iBrightTM software.
Note that images show an overlay of the chemiluminescence signal and the membrane in bright field.

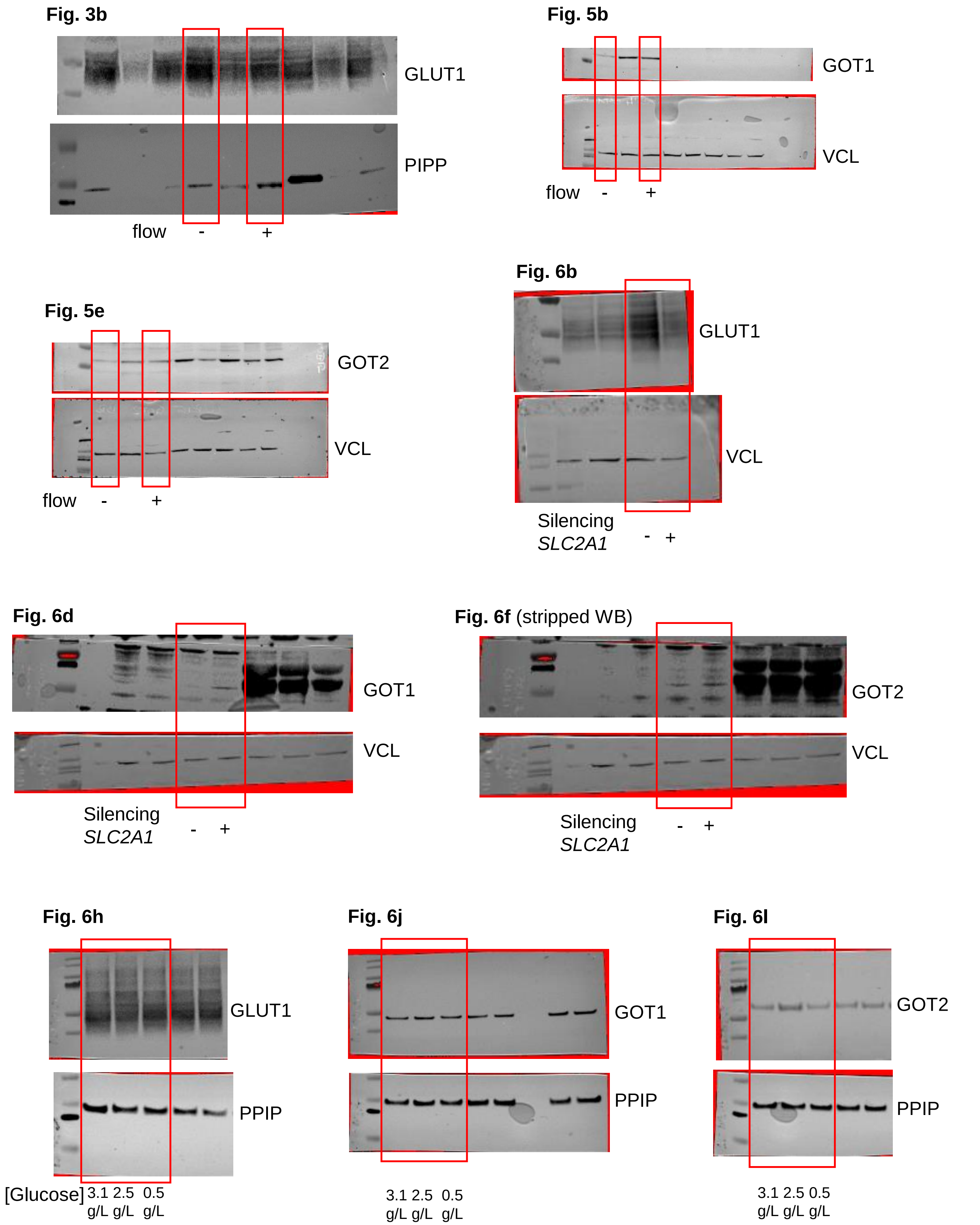

Fig. 7a

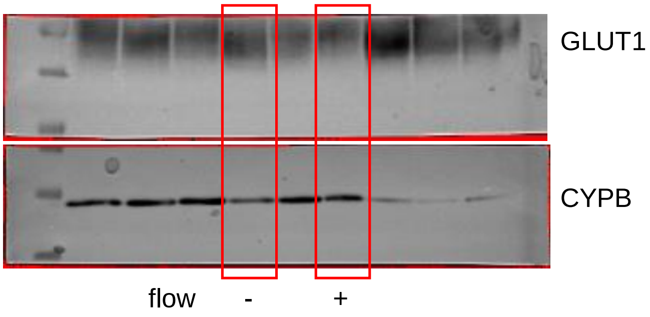

Fig. 7e

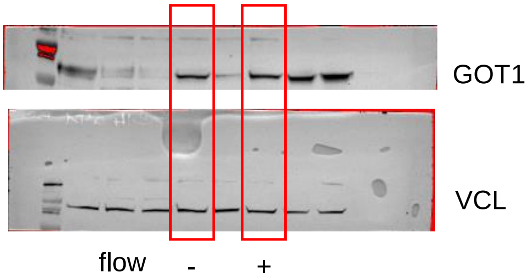

Fig. 7o

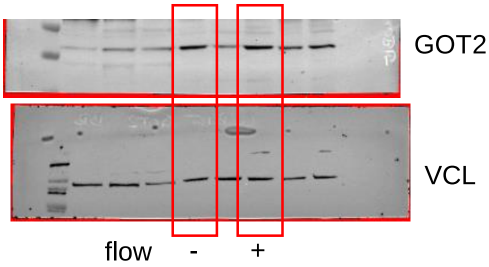

Fig. 8a

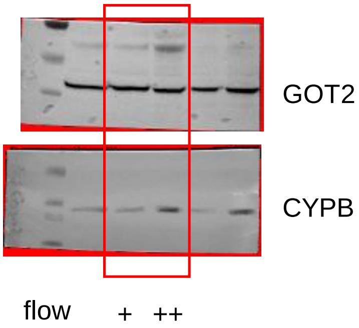

Suppl. Fig. 5b

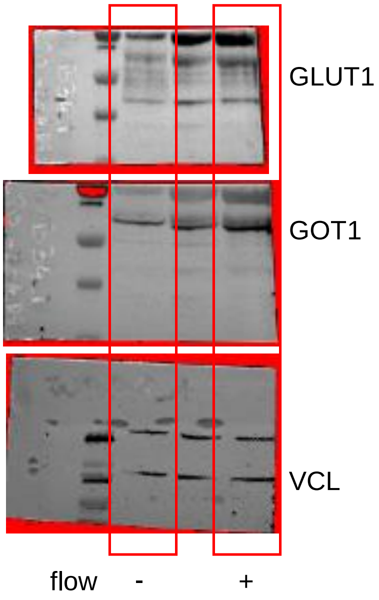

Suppl. Fig. 5i

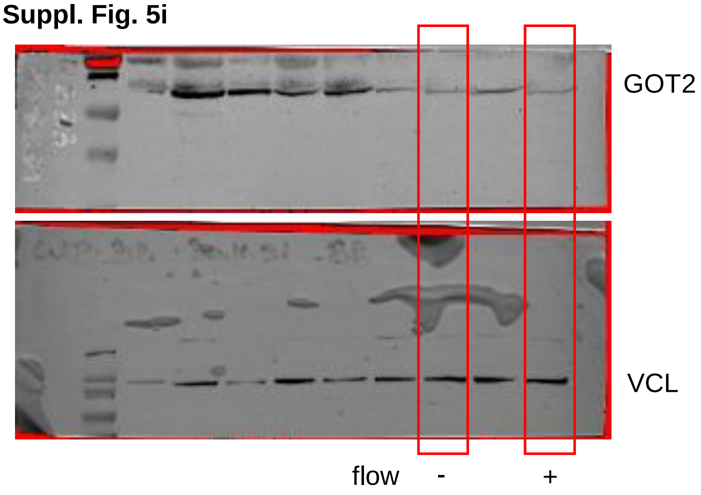

Supplement: Supplementary file 1 — Additional file 1: Figure S1. Validation of cell viability by staining of cleaved caspase 3. First trimester placental villi were stained for cleaved caspase 3 and E-cadherin. A positive control with first trimester villi treated with Staurosporin (2 µM) for 4 h as previously described [64] was used (a, arrowheads indicate caspase 3 positive cells). Placental villi cultured under flow (c) did not show increased caspase 3 activation, when compared to static conditions (b). Figure S2. Syncytiotrophoblast markers in response to fluidic flow. Gene expression patterns (relative rlog values) of differentiated BeWo cells treated either under static conditions (light grey legend) or flow culture (dark grey legend) for 24 h. Figure S3. Key steps in glycolysis. Glycolysis converts glucose to pyruvate (left pathway), while GOT1 and GOT2 convert cysteinesulfinate to pyruvate and taurine (centre path). Cysteine can also be converted to glutathione (right path). Figure S4. Effects of fluidic flow on the glucose uptake and their final processing in trophoblasts. Intracellular uptake of 2-NBDG-Glucose by differentiated BeWo cells either under static or fluidic flow culture (3 ml/min) for 24 h (a). Glucose concentration in supernatant in undifferentiated (DMSO) and differentiated (forskolin) BeWo cells, cultured under static or flow conditions (b). Expression of lactate dehydrogenase subunit B (c, encoded by LDHB) and component X of the pyruvate dehydrogenase (d, PDHX) was analyzed by qPCR. Scale bar in (a) represents 50 µm. Data are presented as mean ± SEM. Experiments were performed with a minimum of three different cell passages. Experiments with placental explants (c and d) were performed with four different placenta samples. Figure S5. Effect of fluidic flow culture on HUVEC. GLUT1 (encoded by SLC2A1) mRNA (a) and protein (b and c) expression, as well as glucose concentration in supernatant (d) and HK2 mRNA expression (e) was analyzed in HUVEC, which were cultured for 24 h eith [file 13578_2023_1114_MOESM1_ESM.zip › Supplementary Figure 9_uncropped Western blots.pdf]
